# Tumor immunosenescence drives breast cancer progression and therapeutic resistance: mechanisms and emerging interventions

**DOI:** 10.3389/fonc.2026.1912627

**Published:** 2026-08-07

**Authors:** Qingran Lei, Xuejun Guo

**Affiliations:** Department of Breast and Thyroid Surgery, First People’s Hospital of Kunming City & Calmette Affiliated Hospital of Kunming Medical University, Kunming, Yunnan, China

**Keywords:** breast cancer, immune checkpoint blockade, immunosenescence, senescence-associated secretory phenotype, senolytics, t cell exhaustion, tumor microenvironment

## Abstract

Breast cancer remains the most frequently diagnosed malignancy and a leading cause of cancer-related mortality among women worldwide. While immune checkpoint inhibitors have transformed treatment for a subset of patients, durable responses remain limited, in part because the tumor immune microenvironment is profoundly shaped by age-associated immune decline—termed immunosenescence. In this review, we critically synthesize how tumor immunosenescence may contribute to breast cancer progression and therapeutic resistance, distinguishing correlative observations from mechanistically established relationships. We examine T cell senescence and exhaustion as mechanistically separable states with distinct therapeutic implications, and evaluate the roles of cancer-associated fibroblasts (CAFs) and tumor-associated macrophages (TAMs) as orchestrators of immunosenescence through SASP production, immune exclusion, and metabolic remodeling. We discuss subtype-specific immunosenescence signatures (TNBC, HER2+, HR+), hormonal and menopausal influences, stage-dependent immune alterations, and BRCA-associated susceptibility. The senescence-associated secretory phenotype (SASP)—comprising IL-6, IL-8, TGF-β, and matrix metalloproteinases—serves as a central mediator linking cellular senescence to immune evasion and metastatic dissemination, though much of the evidence derives from preclinical models. Therapy-induced senescence following chemotherapy and radiotherapy may paradoxically drive relapse through SASP-mediated immunomodulation, a hypothesis that lacks prospective clinical validation. We review clinical evidence that aging is associated with diminished interferon signaling, reduced tumor-infiltrating lymphocyte density, and altered checkpoint blockade efficacy in some but not all studies, acknowledging substantial inter-patient heterogeneity. We highlight recent advances in single-cell and spatial profiling technologies and discuss challenges in biomarker standardization and clinical implementation. Finally, we evaluate emerging interventions—senolytics (navitoclax, dasatinib plus quercetin, fisetin), senomorphics, metabolic rescue via NAD+ repletion, STING agonists, and rational senotherapy-immunotherapy combinations—emphasizing that most remain at preclinical or early clinical stages. We conclude that targeting tumor immunosenescence represents a promising but incompletely validated therapeutic axis for enhancing anti-tumor immunity in breast cancer.

## 1 Introduction

Breast cancer is the most commonly diagnosed cancer in women, accounting for approximately 2.3 million new cases and 685,000 deaths globally each year (1). Despite substantial advances in early detection, surgical resection, radiation therapy, and systemic treatments—including endocrine therapy, HER2-targeted agents, chemotherapy, and more recently immune checkpoint inhibitors (ICIs)—a considerable proportion of patients eventually develop therapeutic resistance and metastatic disease. The tumor immune microenvironment (TIME) has emerged as a critical determinant of treatment response and clinical outcome, yet our understanding of how host-level factors, particularly aging, shape the functional competence of anti-tumor immunity remains incomplete.

Aging is the single greatest risk factor for cancer development. Epidemiological data demonstrate that the incidence of breast cancer rises sharply after the age of 50, and the median age at diagnosis is approximately 62 years (2). Aging is accompanied by a progressive deterioration of the immune system, collectively termed immunosenescence, which encompasses thymic involution with reduced naive T cell output, contraction of the T cell receptor (TCR) repertoire, accumulation of terminally differentiated and senescent T cells, impaired natural killer (NK) cell cytotoxicity, dysregulated myeloid cell function, and chronic low-grade systemic inflammation—often referred to as “inflammaging” (3, 4). These age-related immune alterations are associated with compromised immune surveillance against nascent tumors and may fundamentally reshape the established tumor microenvironment in ways that promote immune escape and therapeutic resistance. However, it is important to acknowledge that much of the evidence linking immunosenescence to breast cancer outcomes remains correlative, and the extent to which immune aging causally drives—rather than merely accompanies—tumor progression and therapeutic resistance varies considerably across studies, subtypes, and patient populations.

The intersection between immunosenescence and breast cancer biology has become an area of intense investigation. Several lines of evidence—primarily from preclinical models and retrospective clinical analyses—suggest that immune aging may actively contribute to tumor progression, though the strength of evidence differs across mechanistic domains. First, senescent CD8+ T cells accumulate in the breast tumor microenvironment, where they exhibit reduced proliferative capacity and cytotoxic function while paradoxically secreting pro-inflammatory mediators that may sustain tumor-promoting inflammation (5). Second, interferon (IFN) signaling, a cornerstone of anti-tumor immunity, has been reported to be progressively diminished with age in both murine breast cancer models and a subset of human triple-negative breast cancer (TNBC) patients, correlating with impaired ICI efficacy in preclinical models, though the clinical evidence remains limited in sample size and requires independent replication (6). Third, therapy-induced senescence (TIS), a cellular response to genotoxic chemotherapy and radiotherapy, generates a reservoir of senescent tumor and stromal cells that secrete SASP factors which may be capable of reprogramming the immune microenvironment toward an immunosuppressive state, thereby potentially facilitating tumor relapse (7, 8). This concept, while mechanistically compelling and supported by murine studies, has not yet been prospectively validated in breast cancer patients.

In this review, we provide a critical synthesis of the mechanisms by which tumor immunosenescence is proposed to drive breast cancer progression and therapeutic resistance. We adopt a systematic approach, examining (i) the cellular compartments of immunosenescence—T cells, NK cells, B cells, CAFs, TAMs, and myeloid cells—and their functional consequences in the breast cancer microenvironment; (ii) the molecular architecture of SASP and its role in immune evasion; (iii) breast cancer-specific aspects of immunosenescence, including subtype-specific signatures, hormonal influences, and stage-dependent immune alterations; (iv) the clinical evidence linking immunosenescence to breast cancer prognosis and immunotherapy outcomes with explicit discussion of conflicting findings and study limitations; and (v) emerging therapeutic strategies designed to reverse, bypass, or exploit immunosenescence for therapeutic benefit framed with appropriate caution regarding their developmental stage. Throughout, we emphasize findings that have been validated in patient-derived samples or clinically relevant model systems, explicitly distinguish between preclinical and clinical evidence, and identify knowledge gaps that warrant further investigation.

## 2 Cellular and molecular mechanisms of tumor immunosenescence in breast cancer

### 2.1 T cell senescence and exhaustion: distinct but interacting states

T cells constitute the principal effector arm of adaptive anti-tumor immunity. In the breast cancer microenvironment, tumor-infiltrating CD8+ T cells frequently exhibit features of either exhaustion or senescence—two mechanistically distinct dysfunctional states that are often conflated in the literature but possess fundamentally different molecular underpinnings, functional consequences, and therapeutic implications. Exhaustion is a state of progressive functional impairment driven by chronic antigen stimulation and characterized by sustained expression of co-inhibitory receptors, hierarchical loss of effector functions, and distinct epigenetic remodeling driven by the transcription factor TOX. In contrast, senescence is a more durable—often irreversible—cell cycle arrest triggered by DNA damage, telomere attrition, oxidative stress, or metabolic collapse, accompanied by a senescence-associated secretory phenotype (SASP) and driven by DNA damage response pathways (ATM, ATR, p53/p21, p16INK4a). Although these states can coexist in the same tumor and may mutually reinforce each other, maintaining their conceptual and mechanistic distinction is essential for rational therapeutic design: reversing exhaustion requires relieving inhibitory receptor signaling (e.g., immune checkpoint blockade), whereas addressing senescence requires either eliminating senescent cells (senolysis), suppressing their secretome (senomorphism), or metabolically rescuing them.

Markers and Drivers of T Cell Senescence. Senescent T cells are characterized by the loss of CD28 expression, re-expression of CD45RA (yielding a CD28^-^CD45RA^+^ terminally differentiated effector memory phenotype), shortened telomeres, elevated expression of the cyclin-dependent kinase inhibitors p16^INK4a and p21^CIP1, and increased senescence-associated β-galactosidase (SA-β-gal) activity (9, 10). These cells also accumulate DNA damage foci marked by phosphorylated histone H2AX (γH2AX) and exhibit mitochondrial dysfunction with elevated reactive oxygen species (ROS) production (10). Critically, senescent T cells are not inert; they acquire a senescence-associated secretory phenotype (SASP) that includes the secretion of IL-6, IL-8, TNF-α, and IFN-γ, which can paradoxically promote tumor cell proliferation and epithelial-mesenchymal transition (EMT). A defining feature of senescent T cells—and one that distinguishes them most clearly from exhausted T cells—is their stable proliferative arrest: they do not re-enter the cell cycle even upon TCR stimulation, whereas exhausted T cells retain some proliferative capacity, particularly in early exhaustion states.

In breast cancer, single-cell transcriptomic analyses have identified a distinct interferon-stimulated gene (ISG)-expressing CD8^+^ T cell subset that displays hallmark features of senescence. Fu and colleagues demonstrated that in early-stage TNBC, ISG^+^CD8^+^ T cells are enriched in tumors resistant to anti-PD-1 immunotherapy (5). Mechanistically, type I interferon produced by HLA-DR^+^ monocytes activates the IRF9–PARP signaling axis in CD8^+^ T cells, leading to excessive consumption of nicotinamide adenine dinucleotide (NAD^+^), a critical cofactor for cellular metabolism and DNA repair. NAD^+^ depletion impairs glycolysis and mitochondrial respiration, driving CD8^+^ T cells into a senescent state characterized by reduced cytotoxicity and failure to respond to PD-1 blockade. This mechanism establishes a direct link between innate immune signaling, metabolic collapse, and T cell senescence in the breast cancer TIME (5). This study provides one of the most detailed mechanistic characterizations of T cell senescence in human breast cancer to date; however, it was conducted in a specific clinical context (early-stage TNBC) and its generalizability to other breast cancer subtypes, disease stages, and patient populations remains to be established.

T Cell Exhaustion in Breast Cancer. Exhausted T cells, in contrast to senescent cells, retain some proliferative potential but exhibit a hierarchical loss of effector functions—IL-2 production, TNF-α secretion, and ultimately cytotoxicity—accompanied by sustained upregulation of multiple co-inhibitory receptors including PD-1, TIM-3, LAG-3, CTLA-4, and TIGIT (11) Exhaustion is an epigenetically imprinted program driven by chronic TCR stimulation and sustained expression of the transcription factor TOX, which distinguishes it at the molecular level from senescence, where DNA damage response pathways predominate. Functionally, exhausted T cells can be at least partially reinvigorated by checkpoint blockade, whereas truly senescent T cells are refractory to PD-1/PD-L1 inhibition alone. Single-cell RNA sequencing of TNBC specimens has revealed extensive intratumoral heterogeneity in exhaustion states, with distinct subpopulations co-expressing PD-1, TIM-3, and LAG-3 that correlate with more advanced exhaustion and worse prognosis (12). In tumor-draining lymph nodes of breast cancer patients, CD8^+^ T cells exhibit exhaustion-related phenotypes with elevated PD-1, TIM-3, and CTLA-4 expression, indicating that exhaustion is initiated early in the anti-tumor immune response and extends beyond the primary tumor site (13). However, these studies are largely observational and do not establish whether exhaustion marker expression directly causes poor outcomes or simply reflects higher antigen burden in more aggressive tumors.

The relationship between senescence and exhaustion is complex and bidirectional. Chronic T cell receptor stimulation, a driver of exhaustion, also induces DNA damage and metabolic stress that can trigger senescence. Conversely, the SASP of senescent T cells contains factors such as IL-6 and TNF-α that can promote bystander T cell exhaustion through sustained inflammatory signaling. This creates a feed-forward loop in which senescent T cells in the TIME actively contribute to the functional impairment of incoming effector T cells. However, direct evidence for this feed-forward loop operating in human breast cancer is currently lacking, and most supporting data derive from *in vitro* co-culture systems and murine models.

CD4^+^ T Cell Immunosenescence and Regulatory T Cell Accumulation. CD4^+^ T helper cells are also profoundly affected by immunosenescence. Aging is associated with a shift from T helper 1 (Th1)- and Th17-dominated responses toward a Th2-polarized phenotype, reducing the capacity for effective anti-tumor immunity (14). Moreover, the thymic output of naive CD4^+^ T cells declines with age, while the peripheral accumulation of FOXP3^+^ regulatory T cells (Tregs) increases (15). In breast cancer, elevated intratumoral Treg density has been consistently associated with poor prognosis, particularly in hormone receptor-positive (HR^+^) and HER2-enriched subtypes (16). A recent study demonstrated that the frequency of CD4^+^FOXP3 exon 2-positive Tregs serves as an independent predictor of breast cancer prognosis and survival (17). Mechanistically, Tregs in the aged breast cancer microenvironment suppress effector T cell function through multiple mechanisms including the secretion of IL-10 and TGF-β, consumption of IL-2, and direct cytolysis via granzyme B and perforin (18). The relative enrichment of Tregs over effector T cells with advancing age tilts the immune balance toward tolerance and contributes to the impaired efficacy of ICIs in older patients.

### 2.2 NK cell senescence and functional impairment

Natural killer (NK) cells are innate lymphocytes that recognize and eliminate tumor cells without prior sensitization. Their activity is governed by the balance between signals from activating receptors (including NKG2D, NKp30, NKp44, NKp46, and DNAM-1) and inhibitory receptors (including killer immunoglobulin-like receptors [KIRs] and NKG2A). In breast cancer, NK cell function is progressively impaired through multiple mechanisms, many of which intersect with the immunosenescence program.

Mamessier and colleagues provided seminal evidence that tumor-infiltrating NK cells in breast cancer patients exhibit a profound functional defect characterized by the downregulation of activating receptors NKG2D, NKp30, DNAM-1, and CD16, coupled with upregulation of the inhibitory receptor NKG2A (19). This phenotypic shift is driven by tumor-derived soluble factors, most notably TGF-β1, which acts as a master regulator of NK cell dysfunction in the breast cancer TIME. Critically, the degree of NKG2D downregulation on tumor-infiltrating NK cells correlated with the Nottingham Prognostic Index, a measure of tumor aggressiveness, and patients who achieved long-term remission (>5 years) after curative surgery showed restoration of normal NK receptor expression profiles (19). These findings demonstrate that NK cell dysfunction in breast cancer is both clinically relevant and, importantly, reversible upon tumor removal.

Additional factors in the breast cancer microenvironment further compound NK cell impairment. Tumor-derived prostaglandin E2 (PGE_2_), soluble MHC class I chain-related molecules (sMICA and sMICB) shed via matrix metalloproteinases, and hypoxia all contribute to the downregulation of NKG2D and the suppression of NK cell cytotoxicity (20). In obese breast cancer patients, lipid droplet accumulation in NK cells further impairs IFN-γ production and perforin/granzyme B-mediated killing, identifying metabolic dysfunction as an additional layer of NK cell immunosenescence (20). The cumulative effect is a severely compromised NK cell compartment that fails to eliminate tumor cells and, through altered cytokine secretion, may actively contribute to an immunosuppressive TIME.

Distinguishing NK Exhaustion, Anergy, and Senescence. It is important to distinguish between the related but mechanistically distinct states of NK cell dysfunction. Exhausted NK cells exhibit progressive functional loss with upregulation of PD-1, TIGIT, and TIM-3, driven by chronic activating receptor stimulation. Anergic NK cells are functionally hyporesponsive without overt inhibitory receptor upregulation. Senescent NK cells, in contrast, display permanent proliferative arrest, telomere shortening, DNA damage accumulation, and SASP secretion (21). In the breast cancer TIME, these states likely coexist and may represent a continuum of dysfunction rather than discrete categories. However, the relative contribution of each state to impaired tumor control, and whether age preferentially drives NK senescence versus exhaustion, has not been systematically investigated. Furthermore, the field lacks robust, consensus markers to operationally distinguish senescent NK cells from exhausted NK cells in human tumor specimens, which limits the interpretability and reproducibility of studies in this area.

### 2.3 Myeloid cell dysfunction in the aged tumor microenvironment

Myeloid cells—including tumor-associated macrophages (TAMs), myeloid-derived suppressor cells (MDSCs), and dendritic cells (DCs)—constitute a major fraction of the immune infiltrate in breast tumors and play pivotal roles in shaping the immune landscape. Aging profoundly alters myelopoiesis, biasing hematopoietic stem cell differentiation toward the myeloid lineage at the expense of lymphoid progenitors, a phenomenon termed “myeloid skewing.” This age-associated shift results in the accumulation of myeloid cells with altered functional properties in both the periphery and the tumor microenvironment (22).

Tumor-Associated Macrophages. TAMs in breast cancer are predominantly polarized toward an M2-like immunosuppressive phenotype characterized by the expression of CD163, IL-10, TGF-β, and arginase-1. These M2-polarized macrophages promote tumor progression through multiple mechanisms: (i) secretion of VEGF and matrix metalloproteinases that facilitate angiogenesis and tissue remodeling; (ii) production of IL-10 and TGF-β that suppress effector T cell function and promote Treg expansion; and (iii) expression of PD-L1 that directly inhibits T cell activity through the PD-1 axis (22). Single-cell transcriptomic analyses of TNBC specimens have confirmed the significant enrichment of M2 macrophages alongside exhausted CD8^+^ T cells, suggesting a coordinated immunosuppressive program (12). In aged hosts, macrophage phagocytic capacity and antigen-presenting function are diminished, while the M2 polarization bias is enhanced, further tilting the TIME toward immune tolerance (23). However, it is important to note that the M1/M2 dichotomy is an oversimplification; TAMs exist along a continuous spectrum of activation states. Moreover, the causal relationship between age-related TAM dysfunction and immunosenescence has not been formally established—it remains possible that TAM alterations are a consequence, rather than a cause, of T cell senescence in the aging TIME.

Myeloid-Derived Suppressor Cells. MDSCs are a heterogeneous population of immature myeloid cells that expand dramatically in cancer and with aging. They are broadly classified into monocytic MDSCs (M-MDSCs) and polymorphonuclear MDSCs (PMN-MDSCs), both of which are potent suppressors of T cell and NK cell function. In breast cancer, MDSC accumulation in peripheral blood and tumor tissue correlates with disease stage, metastatic burden, and poor prognosis (22). MDSCs suppress anti-tumor immunity through the production of arginase-1, inducible nitric oxide synthase (iNOS), reactive oxygen species, and immunosuppressive cytokines including IL-10 and TGF-β. The age-related expansion of MDSCs represents a has been proposed to represent a critical link between systemic immunosenescence and local immune suppression in the breast cancer TIME (3), though most evidence derives from murine models and cross-sectional human studies that demonstrate association rather than causation.

Dendritic Cell Impairment. Dendritic cells are professional antigen-presenting cells essential for priming naive T cells and initiating adaptive anti-tumor immune responses. Aging is associated with reduced DC numbers, impaired antigen uptake and processing capacity, decreased expression of co-stimulatory molecules (CD80, CD86), and diminished IL-12 production (23). In breast cancer patients, circulating and tumor-infiltrating DCs acquire an immature or tolerogenic phenotype, characterized by low MHC class II and co-stimulatory molecule expression, that promotes T cell anergy rather than activation (24). The functional impairment of DCs with age compromises the initiation of productive anti-tumor T cell responses and may contribute to the limited efficacy of DC-based vaccines in older breast cancer patients. Nevertheless, studies directly comparing DC function in young versus older breast cancer patients using standardized functional assays are scarce, and the magnitude of age-related DC impairment in the specific context of breast cancer remains poorly quantified.

### 2.4 B cell and tertiary lymphoid structure alterations

B cells and the tertiary lymphoid structures (TLS) they organize have emerged as important determinants of anti-tumor immunity and immunotherapy response. TLS are ectopic lymphoid aggregates that form in non-lymphoid tissues during chronic inflammation and contain B cell follicles, germinal centers, T cell zones, and dendritic cells. Their presence in tumors has been associated with favorable prognosis and improved ICI responses across multiple cancer types, including breast cancer (25).

In breast cancer, mature TLS—defined by the presence of CD21^+^ follicular dendritic cell networks and germinal center reactions—are found in approximately 5–6% of cases and are independently associated with improved recurrence-free survival and better ICI response (26). Single-cell transcriptomic analysis has revealed substantial B cell heterogeneity within breast tumors, with TLS-associated B cells exhibiting markers of germinal center reactions, somatic hypermutation, and clonal expansion indicative of ongoing humoral immunity (27). The landmark study by Helmink and colleagues demonstrated that B cells and TLS promote immunotherapy response through the generation of tumor-reactive antibodies, antigen presentation to T cells, and local cytokine production that sustains effector T cell function (28).

However, how immunosenescence affects B cell function and TLS formation in breast cancer is not well understood. Aging is associated with reduced B cell lymphopoiesis in the bone marrow, diminished antibody diversity, impaired affinity maturation, and accumulation of age-associated B cells (ABCs)—a transcriptionally distinct subset that produces TNF-α and may contribute to chronic inflammation (4). The relevance of these age-related B cell alterations to anti-tumor immunity and TLS biology in breast cancer represents a significant knowledge gap. Whether TLS formation, maintenance, and function decline with age, and whether this contributes to reduced ICI efficacy in older patients, remains to be investigated. The few studies that have examined TLS in breast cancer have not systematically analyzed the effect of patient age on TLS density, maturation status, or prognostic significance. This represents an important area for future investigation, particularly given the growing interest in TLS as a predictive biomarker for immunotherapy.

### 2.5 Thymic involution and impaired central immune surveillance

Thymic involution—the progressive, age-dependent shrinkage and functional decline of the thymus—is one of the earliest and most consequential manifestations of immunosenescence. Beginning in early adulthood, the thymic epithelium is progressively replaced by adipose tissue, resulting in a logarithmic decline in the production of naive T cells and a corresponding contraction of the peripheral TCR repertoire (29). By the sixth decade of life, thymic output of naive T cells is reduced by more than 90% compared with young adulthood, severely limiting the capacity to generate *de novo* immune responses against novel tumor antigens (29).

The consequences of thymic involution for cancer immune surveillance are profound. A restricted TCR repertoire diminishes the probability of recognizing tumor neoantigens generated by somatic mutations, compromising the breadth and effectiveness of adaptive anti-tumor immunity (29). Additionally, the involuted thymus exhibits a relative increase in the generation of thymic Tregs (tTregs), which, together with age-related peripheral Treg accumulation, reinforces immune suppression in the tumor microenvironment. Chemotherapy-induced acute thymic involution—a common consequence of cytotoxic cancer treatment—further exacerbates this problem by creating a potential reservoir for dormant disseminated tumor cells, which can later seed metastatic relapse (29).

Experimental evidence supports the clinical relevance of thymic function to breast cancer outcomes. Palmer and colleagues used mathematical modeling to demonstrate that age-related decline in thymic T cell production is a major risk factor for cancer development, independent of mutational burden (30). Conversely, strategies that enhance thymic function or compensate for reduced thymic output through adoptive T cell transfer or TCR engineering are being explored as potential interventions to restore immune competence in aged cancer patients (29).

### 2.6 Cancer-associated fibroblasts and tumor-associated macrophages as orchestrators of immunosenescence

Cancer-associated fibroblasts (CAFs) and tumor-associated macrophages (TAMs) are the most abundant non-malignant stromal cell populations in the breast tumor microenvironment, and their roles in shaping immune function and therapeutic response are increasingly appreciated. However, their specific contributions to tumor immunosenescence have received relatively limited attention and warrant dedicated discussion.

#### 2.6.1 CAFs and Immunosenescence

CAFs are heterogeneous mesenchymal cells that arise from resident fibroblasts, bone marrow-derived mesenchymal stem cells, adipocyte precursors, or endothelial cells through mesenchymal transition. In breast cancer, multiple CAF subpopulations have been identified through single-cell analyses, including inflammatory CAFs (iCAFs), myofibroblastic CAFs (myCAFs), and antigen-presenting CAFs (apCAFs), each with distinct transcriptional programs and immune-modulatory functions. With advancing age, tissue-resident fibroblasts accumulate hallmarks of cellular senescence—including DNA damage foci, elevated p16INK4a and p21CIP1, and SA-β-gal activity—and transition toward a pro-inflammatory SASP-producing state. Senescent CAFs are a dominant source of SASP factors in the breast TME, secreting high levels of IL-6, IL-8, CXCL1, CXCL8, CCL2, TGF-β, VEGF, and multiple matrix metalloproteinases (MMP-1, MMP-3, MMP-9). These factors collectively promote tumor cell proliferation, epithelial-mesenchymal transition (EMT), angiogenesis, and extracellular matrix remodeling. Importantly, the SASP of senescent CAFs actively remodels the immune landscape by recruiting MDSCs and M2-polarized macrophages through CCL2 and CXCL1 gradients, suppressing CD8+ T cell cytotoxicity through TGF-β, excluding T cells from tumor nests through dense extracellular matrix deposition, and promoting FOXP3+ Treg differentiation. A particularly striking finding is that SASP-conditioned media from senescent fibroblasts can induce NLRP3 inflammasome/caspase-1/gasdermin D-dependent pyroptotic cell death in normal mammary epithelial cells, whereas oncogenically transformed cells are relatively protected—a mechanism that may selectively eliminate normal epithelial competitors while sparing established tumor cells (35).

Despite these mechanistic insights, several important questions remain unanswered. The extent to which CAF senescence is driven by chronological aging of the host versus tumor-derived signals versus prior therapeutic exposure (chemotherapy, radiation) is unclear. Single-cell analyses have revealed substantial CAF heterogeneity in breast cancer, but the relative abundance and functional significance of senescent CAF subsets across different breast cancer subtypes, patient ages, and treatment histories have not been systematically catalogued. Furthermore, whether senolytic agents effectively eliminate senescent CAFs in human breast tumors, and whether CAF-targeted senolysis improves anti-tumor immunity in patients, remains to be tested clinically.

#### 2.6.2 TAMs and Immunosenescence

TAMs represent up to 50% of the cellular mass in some breast tumors and are key integrators of the relationship between aging, inflammation, and immune suppression. Aging promotes myeloid skewing of hematopoiesis and enhances M2-like polarization of TAMs. Beyond these cell-intrinsic changes, aged TAMs exhibit diminished phagocytic capacity, reduced antigen-presenting capacity, increased SASP factor production (IL-6, IL-8, TNF-α, CCL2), contributing to systemic inflammaging that drives bystander T cell senescence and exhaustion, and enhanced PD-L1 expression. Critically, TAMs and CAFs engage in bidirectional crosstalk that amplifies immunosenescence: CAF-derived IL-6 and CCL2 promote monocyte recruitment and M2 polarization, while TAM-derived TGF-β and PDGF drive CAF activation and senescence. However, direct experimental evidence for this loop operating in human breast tumors is limited, and its relative importance compared with tumor cell-intrinsic drivers of immunosenescence has not been established.

A key unresolved question is the relationship between TAMs and therapy-induced senescence. Chemotherapy and radiotherapy induce senescence not only in tumor cells but also in TAMs themselves, potentially converting them into SASP-producing cells that exacerbate post-treatment immune suppression. The clinical significance of CAF/TAM-driven immunosenescence in breast cancer is supported primarily by correlative evidence. Prospective studies that quantify senescent CAF and TAM burden using validated, consensus markers of senescence are needed to establish the contribution of stromal cell senescence to clinical immunosenescence.

### 2.7 Metabolic and epigenetic distinctions among exhausted, senescent, and aged T cells

The metabolic and epigenetic landscapes of exhausted, senescent, and aged T cells exhibit both shared features and critical differences that carry distinct therapeutic implications. Clarifying these distinctions is essential, as therapeutic strategies that are effective for one dysfunctional state may be ineffective or even counterproductive for another.

Metabolic distinctions. Exhausted T cells are characterized by suppressed glycolysis and glutaminolysis, reduced mitochondrial oxidative phosphorylation (OXPHOS), and diminished nutrient uptake driven in part by persistent PD-1 signaling. Senescent T cells, by contrast, exhibit a distinct metabolic profile driven by chronic DNA damage signaling and mitochondrial dysfunction. In breast cancer, sustained type I IFN signaling in CD8+ T cells activates the IRF9-PARP axis, leading to excessive consumption of NAD+—a critical coenzyme for glycolysis, the TCA cycle, and oxidative phosphorylation (5). NAD+ depletion results in a profound bioenergetic crisis that is mechanistically distinct from the PD-1-driven metabolic suppression of exhausted cells and is irreversible by checkpoint blockade alone. Aged T cells occupy an intermediate metabolic state: they exhibit reduced mitochondrial mass and membrane potential, increased mitochondrial ROS production, and a shift toward fatty acid oxidation, but retain some metabolic flexibility and can respond partially to metabolic interventions such as AMPK activation or mTOR inhibition.

Epigenetic distinctions. Exhaustion is associated with a distinct chromatin accessibility landscape driven by the transcription factor TOX, which cooperates with NR4A family members. The exhaustion epigenome includes open chromatin at loci encoding co-inhibitory receptors (PDCD1, HAVCR2, LAG3, CTLA4) and repression of loci associated with T cell memory (TCF7, IL7R). Early exhaustion states are reversible, whereas late exhaustion is epigenetically fixed and resistant to checkpoint blockade. T cell senescence is epigenetically governed by DNA damage response pathways: activation of p53/p21CIP1 and p16INK4a/RB pathways; formation of senescence-associated heterochromatin foci (SAHF); histone modifications (H3K9me3, macroH2A1) at SASP gene loci; and LINE-1 retrotransposon de-repression activating the cGAS-STING pathway. Critically, the epigenetic changes of senescence are generally more stable and less reversible than those of exhaustion—a distinction with major therapeutic implications.

Therapeutic implications. These metabolic and epigenetic distinctions underscore why conflating exhaustion and senescence can lead to suboptimal therapeutic choices. In a tumor containing predominantly exhausted T cells, checkpoint blockade is a rational first-line strategy. In a tumor containing predominantly senescent T cells, checkpoint blockade is unlikely to be effective, and alternatives such as NAD+ repletion, senolytic therapy, or adoptive cell transfer should be prioritized. The development of biomarkers that accurately quantify the relative contributions of each dysfunctional state in individual patients is an urgent translational priority.

### 2.8 Hallmarks and primary pathways of tumor cell senescence

A comprehensive understanding of tumor immunosenescence requires delineating the hallmarks of senescence in tumor cells themselves, as these senescent tumor cells are both targets of immune surveillance and sources of SASP-mediated immune modulation. The core hallmarks of cellular senescence in breast tumor cells include: (i) stable proliferative arrest, mediated by the p53/p21CIP1 and p16INK4a/RB tumor suppressor pathways; (ii) macromolecular damage, including telomere attrition, persistent DNA damage foci (γH2AX, 53BP1), and protein aggregates; (iii) metabolic reprogramming, characterized by mitochondrial dysfunction with elevated ROS, NAD+ depletion, and a shift toward fatty acid oxidation and glutamine dependency; (iv) the senescence-associated secretory phenotype (SASP), transcriptionally governed by NF-κB, C/EBPβ, JAK2/STAT3, p38 MAPK, and NOTCH1, with context-dependent contributions from cGAS-STING signaling; (v) epigenetic remodeling, including SAHF formation, global chromatin compaction changes, and activation of endogenous retrotransposons (LINE-1, Alu); (vi) upregulation of anti-apoptotic networks, particularly BCL-2 family proteins (BCL-2, BCL-XL, BCL-W), which create a therapeutic vulnerability exploitable by senolytic agents; (vii) lysosomal expansion with increased SA-β-gal activity; and (viii) altered cell surface proteome, including upregulation of PD-L1, CD80, Galectin-9, and in some contexts uPAR and DPP4.

**Figure 1:**
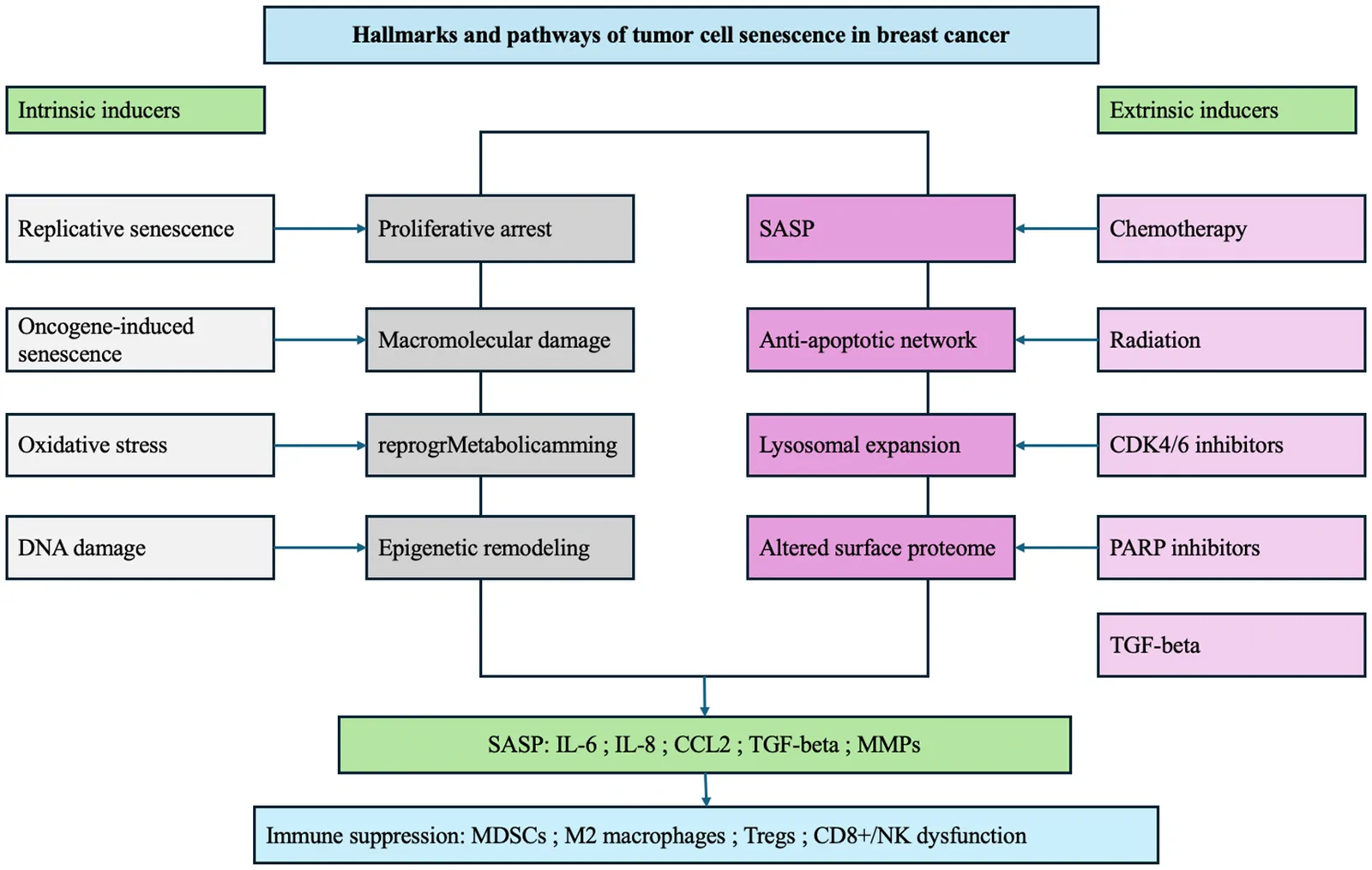
Hallmarks and pathways of tumor cell senescence in breast cancer. Tumor cell senescence in breast cancer can be initiated by intrinsic and extrinsic stressors. Intrinsic inducers, including replicative exhaustion, oncogene activation, oxidative stress, and DNA damage, trigger proliferative arrest through multiple mechanisms, including macromolecular damage accumulation, metabolic reprogramming, and epigenetic remodeling. Extrinsic therapeutic stresses, such as chemotherapy, radiotherapy, CDK4/6 inhibitors, PARP inhibitors, and TGF-β signaling activation, induce or reinforce senescent phenotypes. Senescent tumor cells exhibit characteristic features, including senescence-associated secretory phenotype (SASP), activation of anti-apoptotic networks, lysosomal expansion, and altered surface proteomic profiles. SASP components, including IL-6, IL-8, CCL2, TGF-β, and matrix metalloproteinases (MMPs), reshape the tumor microenvironment and promote immune suppression through the recruitment or activation of myeloid-derived suppressor cells (MDSCs), M2 macrophages, regulatory T cells (Tregs), and dysfunction of CD8+ T cells and natural killer (NK) cells.

The primary pathways driving senescence in breast tumor cells include intrinsic pathways (replicative senescence, oncogene-induced senescence, oxidative stress-induced senescence, and DNA damage-induced senescence) and extrinsic pathways (therapy-induced senescence from genotoxic chemotherapy, radiation, CDK4/6 inhibitors, and PARP inhibitors; and TGF-β-driven paracrine senescence) (**Figure 1**). **Table 1** provides a comprehensive overview of the cellular and molecular mechanisms of immunosenescence across all immune and stromal compartments in the breast cancer microenvironment.

**Table 1:** Cellular and molecular mechanisms of immunosenescence in the breast cancer microenvironment.

Cell type	Senescent/dysfunctional phenotype	Key molecular markers	SASP/secreted mediators	Clinical relevance in breast cancer
CD8^+^ T cells	Loss of CD28, re-expression of CD45RA (CD28^-^CD45RA^+^ TEMRA); telomere shortening; DNA damage (γH2AX^+^); mitochondrial dysfunction with elevated ROS; metabolic exhaustion via NAD^+^ depletion	p16^INK4a, p21^CIP1, SA-β-gal, γH2AX, PD-1, TIM-3, LAG-3, CTLA-4, TIGIT; ISG signature (IRF9–PARP axis) [RED: distinguishing exhaustion (TOX-driven, checkpoint-reversible) from senescence (DDR-driven, checkpoint-irreversible)]	IL-6, IL-8, TNF-α, IFN-γ	ISG^+^CD8^+^ T cells enriched in anti-PD-1-resistant early TNBC; NAD^+^ depletion driven by monocyte-derived type I IFN predicts ICI failure (5)
CD4^+^ T cells/Tregs	Th1/Th17 → Th2 polarization shift; reduced naive CD4^+^ output; increased FOXP3^+^ Treg accumulation with age; enhanced suppressive function	FOXP3, CTLA-4, GITR, CD25, FOXP3 exon 2	IL-10, TGF-β, granzyme B, perforin	Elevated intratumoral Treg density associated with poor prognosis in HR^+^ and HER2-enriched subtypes (16); CD4^+^FOXP3Exon2^+^ Treg frequency independently predicts survival (17) [RED: paradoxically, higher stromal Treg and PD-L1 associated with improved survival in young (≤40 yr) HR+ patients (42)]
NK cells	Downregulation of activating receptors; upregulation of inhibitory receptors; lipid droplet accumulation in obesity; reduced IFN-γ and perforin/granzyme B	NKG2D↓, NKp30↓, DNAM-1↓, CD16↓; NKG2A↑; PD-1, TIGIT, TIM-3 (exhaustion)	Altered cytokine secretion	NKG2D downregulation correlates with Nottingham Prognostic Index; receptor profile normalizes after curative surgery (>5 yr remission) (19); obesity exacerbates dysfunction (20)
Tumor-associated macrophages (TAMs)	M2-like polarization bias enhanced with age; diminished phagocytosis and antigen presentation [RED: bidirectional crosstalk with CAFs amplifies immunosenescence; TAM senescence post-chemotherapy may exacerbate SASP-mediated immune suppression]	CD163, CD206, Arginase-1, PD-L1	IL-10, TGF-β, VEGF, MMPs	M2 macrophages enriched alongside exhausted CD8^+^ T cells in TNBC (12); age-enhanced M2 polarization promotes immune tolerance (23)
Myeloid-derived suppressor cells (MDSCs)	Age-related expansion of M-MDSCs and PMN-MDSCs; potent T cell and NK cell suppression	CD11b, Gr-1, CD14 (M-MDSC), CD15 (PMN-MDSC)	Arginase-1, iNOS, ROS, IL-10, TGF-β	MDSC accumulation correlates with disease stage, metastatic burden, and poor prognosis (22); key [RED: proposed as] link between systemic immunosenescence and local TIME suppression (3)
Dendritic cells (DCs)	Reduced numbers; impaired antigen uptake/processing; decreased co-stimulatory molecule expression; diminished IL-12; tolerogenic phenotype	CD80↓, CD86↓, MHC-II↓, IL-12↓	Altered cytokine milieu	Tolerogenic DCs promote T cell anergy rather than activation (24); compromises initiation of anti-tumor T cell responses in older patients [RED: magnitude of age-related DC impairment in breast cancer context poorly quantified]
B cells/TLS	Reduced B cell lymphopoiesis with age; diminished antibody diversity; impaired affinity maturation; accumulation of age-associated B cells (ABCs); TLS formation in ~5–6% of breast cancers	CD21^+^ FDC networks, AID, BCL-6 (germinal center); TNF-α (ABCs)	Tumor-reactive antibodies, TNF-α (ABCs)	Mature TLS independently associated with improved recurrence-free survival and better ICI response (26, 28); role of B cell immunosenescence in TLS biology remains largely unexplored [RED: effect of age on TLS density, maturation, and function remains unexplored]
Cancer-associated fibroblasts (CAFs)	Senescent CAFs are dominant SASP source in breast TME; age-associated accumulation of p16+/SA-β-gal+ CAFs; inflammatory (iCAF) and myofibroblastic (myCAF) subsets with distinct immune-modulatory functions	p16INK4a, p21CIP1, SA-β-gal, α-SMA, FAP, PDGFRα/β	IL-6, IL-8, CXCL1, CXCL8, CCL2, TGF-β, VEGF, MMP-1/3/9; drives pyroptotic death of normal mammary epithelial cells via NLRP3/caspase-1/GSDMD (35)	Higher senescent CAF burden associated with older age and worse prognosis in retrospective cohorts; promotes immune exclusion via ECM remodeling and MDSC/Treg recruitment; CAF senescence not systematically examined across subtypes or treatment histories

TEMRA, terminally differentiated effector memory cells re-expressing CD45RA; SA-β-gal, senescence-associated β-galactosidase; ISG, interferon-stimulated gene; FDC, follicular dendritic cell; AID, activation-induced cytidine deaminase; ABC, age-associated B cell.

## 3 The senescence-associated secretory phenotype in the breast cancer microenvironment

### 3.1 SASP composition and signaling networks

Cellular senescence is a state of stable proliferative arrest triggered by diverse stressors including telomere attrition, DNA damage, oncogene activation, oxidative stress, and genotoxic therapy. While senescence serves as a tumor-suppressive mechanism by preventing the replication of damaged cells, senescent cells are metabolically active and secrete a complex mixture of bioactive molecules collectively termed the senescence-associated secretory phenotype (SASP). The SASP includes pro-inflammatory cytokines (IL-6, IL-8, IL-1β), chemokines (CCL2, CCL5, CXCL1, CXCL8), growth factors (TGF-β, VEGF, HGF), matrix metalloproteinases (MMP-1, MMP-3, MMP-9), and extracellular matrix components (31, 32).

The SASP is transcriptionally regulated primarily by the NF-κB and C/EBPβ pathways, with additional contributions from JAK/STAT signaling, p38 MAPK, and NOTCH (31) Emerging evidence also implicates the cGAS-STING pathway, which is activated by cytosolic chromatin fragments and LINE-1 retrotransposons in senescent cells, as an upstream activator of NF-κB and IRF3 that reinforces SASP expression. The mTOR pathway further contributes to SASP production through enhanced translation of SASP factor mRNAs. The composition of the SASP varies depending on the senescence-inducing stimulus, the cell type, and the duration of senescence—a heterogeneity that carries important therapeutic implications but also complicates the development of universal SASP-targeting strategies. In the breast cancer microenvironment, SASP factors are produced by multiple cell types: senescent tumor cells (whether from replicative senescence or therapy-induced senescence), senescent cancer-associated fibroblasts (CAFs), senescent endothelial cells, senescent adipocytes, and senescent immune cells (32, 33) The relative contribution of each cell type to the total SASP burden in human breast tumors has not been systematically quantified and likely varies across subtypes, disease stages, and treatment histories.

Recent work has identified transcriptionally distinct senescent populations in breast tumors. Oesterreich and Aird demonstrated that senescent breast cancer cells upregulate redundant immunoinhibitory proteins, with two distinct populations identified: a CD80-enriched population driven by NF-κB and a PD-L1/Galectin-9-enriched population driven by IFN signaling (34). This heterogeneity suggests that different senescent subpopulations may differentially contribute to immune evasion and that therapeutic strategies may need to target multiple senescent subsets simultaneously. However, this classification was derived from *in vitro* models, and whether these distinct senescent subpopulations exist and remain stable in human breast tumors is not yet known.

Fibroblast-Derived SASP. Cancer-associated fibroblasts are a major source of SASP factors in the breast cancer microenvironment. Senescent CAFs secrete elevated levels of IL-6, IL-8, MMPs, and TGF-β that promote tumor cell proliferation, invasion, EMT, and angiogenesis (33). Notably, SASP-conditioned media from senescent fibroblasts can induce NLRP3 inflammasome/caspase-1/gasdermin D-dependent pyroptotic cell death in normal mammary epithelial cells, whereas oncogenically transformed cells are relatively protected—a mechanism that may selectively eliminate normal epithelial competitors while sparing established tumor cells (35). This finding, while mechanistically intriguing, is based on *in vitro* co-culture experiments and its relevance to the *in vivo* tumor microenvironment remains to be validated.

### 3.2 SASP-mediated immune evasion

The SASP exerts pleiotropic effects on the tumor immune microenvironment, many of which favor immune evasion. SASP factors including IL-6, IL-8, CCL2, and CXCL1 serve as potent chemoattractants for MDSCs and M2-polarized macrophages, driving their recruitment and accumulation in the tumor bed (31). Once recruited, these immunosuppressive myeloid cells are further activated by SASP-derived TGF-β and IL-10, establishing a self-reinforcing cycle of immune suppression. However, direct *in vivo* evidence for this feed-forward loop in breast cancer patients is limited, and the relative importance of SASP-driven myeloid recruitment versus tumor cell-intrinsic chemokine production has not been resolved.

The SASP also directly modulates lymphocyte function. TGF-β, a canonical SASP component, inhibits CD8^+^ T cell proliferation and cytotoxic function, suppresses NK cell activity through the downregulation of NKG2D, and promotes the differentiation of naive CD4^+^ T cells into FOXP3^+^ Tregs (31, 36). IL-6, acting through STAT3, contributes to T cell exhaustion and impairs Th1 differentiation. Furthermore, senescent tumor cells upregulate surface expression of PD-L1, CD80, and Galectin-9, engaging multiple immune checkpoint pathways that directly inhibit T cell activity (34). The relative contribution of each SASP component to overall immune evasion in breast cancer has not been dissected, and it is likely that the dominant mechanisms differ across tumor subtypes and anatomical sites (primary tumor versus metastatic niche).

An important study by Muñoz and colleagues demonstrated that persistent senescent cells deploy targetable mechanisms of immunoevasion that link chemotherapy-resistant cancer to aging (7). Senescent cells evade immune clearance through the upregulation of PD-L1 and the downregulation of MHC class I expression, rendering them resistant to both innate and adaptive immune recognition. This dual mechanism—SASP-mediated recruitment of immunosuppressive cells combined with cell-intrinsic immune evasion—enables senescent cells to persist in tissues and drive tumor recurrence after chemotherapy. Notably, this study was conducted in cell line xenograft models, and whether the same immune evasion mechanisms operate in spontaneously arising senescent cells in immunocompetent hosts with intact immune systems has not been demonstrated.

### 3.3 Therapy-induced senescence: a double-edged sword

Chemotherapy and radiotherapy, mainstays of breast cancer treatment, induce cellular senescence in a substantial fraction of tumor cells and stromal cells. While TIS was historically viewed as a favorable therapeutic outcome—eliminating the proliferative capacity of cancer cells—it is now recognized as a double-edged sword. Senescent cells that persist after treatment secrete SASP factors that, in preclinical models, can promote the survival, stemness, and metastatic capacity of residual non-senescent tumor cells, while simultaneously suppressing anti-tumor immunity (8, 37). However, it is important to emphasize that the relative contribution of TIS to disease relapse in breast cancer patients, as opposed to other mechanisms of treatment failure (e.g., selection of pre-existing resistant clones, tumor cell dormancy, or adaptive metabolic reprogramming), has not been quantified. The TIS-driven relapse hypothesis, while mechanistically plausible and supported by murine studies, currently lacks prospective clinical validation in breast cancer.

In breast cancer, TIS has been documented after treatment with doxorubicin, cyclophosphamide, paclitaxel, radiation, CDK4/6 inhibitors, and PARP inhibitors. The combination of radiation therapy with PARP inhibition (talazoparib) has been shown to induce robust senescence in TNBC models, and the subsequent elimination of senescent cells with the BCL-2/BCL-XL inhibitor navitoclax (ABT-263) dramatically delayed tumor regrowth *in vivo* (38). Similarly, combining venetoclax (a BCL-2-selective inhibitor) with radiotherapy and talazoparib effectively eliminated therapy-induced senescent TNBC cells in immunocompetent mouse models (39). These proof-of-concept studies established the “one-two punch” strategy—senescence induction followed by senolytic clearance—as a promising therapeutic approach for breast cancer. However, it must be noted that these studies were conducted in immunocompetent murine models (E0771 and 4T1), which do not fully recapitulate the complexity of human breast cancer biology, the heterogeneity of senescence responses, or the decades-long time course over which human breast cancers evolve and recur. The translatability of these results to patients remains to be demonstrated in clinical trials.

A critical clinical consideration is that TIS affects not only tumor cells but also normal tissue cells. Chemotherapy and radiotherapy induce senescence in bone marrow hematopoietic stem cells, thymic epithelial cells, and peripheral immune cells, potentially exacerbating pre-existing immunosenescence and creating a more permissive environment for tumor recurrence (8). This systemic toxicity of TIS highlights the need for senolytic strategies that selectively eliminate senescent tumor cells while sparing essential normal tissue compartments.

### 3.4 Major etiological factors in the TME that promote immune senescence

The tumor microenvironment contains a convergence of factors that collectively drive immune cell senescence. We summarize the primary etiological categories below:

#### 3.4.1 Chronic antigenic stimulation

Persistent exposure to tumor antigens drives repeated TCR stimulation in tumor-infiltrating T cells, leading to DNA damage accumulation, telomere attrition, and ultimately replicative senescence. This mechanism is particularly relevant in immunogenic breast cancer subtypes (TNBC, HER2+) with higher mutational burden.

#### 3.4.2 Oxidative stress and metabolic competition

The breast TME is characterized by hypoxia, nutrient deprivation, and elevated ROS from both tumor cells and activated myeloid cells. Tumor cells compete with infiltrating T cells for glucose and glutamine, imposing metabolic stress that accelerates T cell senescence.

#### 3.4.3 Tumor-derived soluble factors

Breast tumor cells secrete TGF-β, IL-10, PGE2, VEGF, adenosine, and soluble MHC class I chain-related molecules (sMICA/sMICB) that directly suppress NK cell and T cell function. TGF-β, in particular, operates as a master regulator of immune dysfunction by downregulating NKG2D, suppressing CD8+ T cell cytotoxicity, and driving Treg differentiation (19, 36).

#### 3.4.4 SASP paracrine signaling

SASP factors from senescent tumor cells, CAFs, and TAMs act in a paracrine manner to induce bystander senescence in previously functional immune cells. IL-6 and IL-8 activate JAK/STAT and NF-κB signaling in recipient cells, promoting both senescence and exhaustion programs.

#### 3.4.5 Therapy-induced genotoxic stress

Chemotherapy and radiotherapy induce DNA damage in tumor-infiltrating immune cells and bone marrow progenitors. The cumulative genotoxic burden across multiple lines of therapy may accelerate immune aging, particularly in older patients with limited regenerative capacity.

#### 3.4.6 Age-related systemic changes

Systemic hallmarks of aging—including thymic involution, myeloid skewing of hematopoiesis, inflammaging, and accumulation of senescent cells in peripheral tissues—create a host environment that is permissive for immune senescence within the TME. Senescent cells in distant tissues (e.g., bone marrow, adipose tissue) secrete SASP factors into the circulation that may act at a distance on tumor-infiltrating immune cells.

#### 3.4.7 Microbiome alterations

Age-related changes in the gut and breast tissue microbiota may contribute to immune senescence through altered microbial metabolite profiles, chronic innate immune activation, and modulation of systemic inflammation (discussed in Section 5.8).

The relative contribution of each factor likely varies across patients, breast cancer subtypes, disease stages, and treatment histories. A systematic, quantitative understanding of how these factors interact to drive immune senescence in individual patients remains a major research priority.

## 4 Clinical significance of immunosenescence in breast cancer

### 4.1 Age-related differences in tumor-infiltrating lymphocytes

Tumor-infiltrating lymphocytes (TILs) have been established as a robust prognostic and predictive biomarker in breast cancer, particularly in TNBC and HER2-enriched subtypes. Higher TIL density is associated with improved pathological complete response (pCR) rates after neoadjuvant chemotherapy and with better long-term survival outcomes (40). However, the relationship between TILs and clinical outcomes is modulated by patient age, reflecting the influence of systemic immunosenescence on the tumor immune microenvironment. Critically, the evidence in this area is largely correlative and derived from retrospective analyses; whether age-related differences in TIL density directly cause differences in treatment outcomes has not been demonstrated prospectively.

Takada and colleagues analyzed TIL density and prognostic factors in 356 breast cancer patients stratified by age (≤45 years versus >60 years) receiving preoperative chemotherapy (41). In TNBC, older patients had significantly lower TIL density compared with younger patients, and this difference was associated with inferior overall survival in the older cohort. In HER2-enriched disease, younger patients with higher TIL density achieved higher pCR rates, consistent with a more immunocompetent tumor microenvironment conferring greater chemotherapy sensitivity. These data suggest that age-dependent immunosenescence directly contributes to treatment resistance in the most immunogenic breast cancer subtypes (41). However, the study did not directly measure immune senescence markers (e.g., p16INK4a, SA-β-gal, telomere length) in TILs, and the observed associations could be confounded by age-related differences in tumor biology, comorbidities, treatment intensity, or other factors independent of immune senescence per se.

In a prospective cohort study of young women (≤40 years) with HR^+^/HER2^-^ breast cancer, Tesch and colleagues used multiplex immunofluorescence to characterize immune cell subtypes and their association with clinical outcomes (42). Paradoxically, within this young cohort, higher immune infiltration was associated with increasing age, suggesting that even within a relatively narrow age range, subtle changes in immune competence can influence the tumor microenvironment. Notably, higher stromal Treg density and PD-L1 expression were associated with improved invasive breast cancer-free survival and distant disease-free survival, challenging the conventional view that Tregs are uniformly detrimental in all breast cancer contexts (42). Salgado, in an accompanying commentary, emphasized that the triad of immunity, age, and luminal breast cancer represents an emerging frontier requiring nuanced biomarker interpretation (43). These findings underscore a broader principle: the prognostic and predictive significance of immune biomarkers in breast cancer is likely context-dependent, varying with patient age, breast cancer subtype, menopausal status, and disease stage. Simple, one-size-fits-all thresholds for TILs or other immune biomarkers may be inadequate for clinical decision-making in age-heterogeneous patient populations.

### 4.2 Breast cancer subtype-specific immunosenescence signatures

Immunosenescence does not manifest uniformly across breast cancer subtypes. The distinct biology of TNBC, HER2-positive, and HR+ breast cancers—each with different mutational burdens, neoantigen loads, and baseline immune infiltration levels—creates subtype-specific contexts in which immune aging operates.

#### 4.2.1 Triple-negative breast cancer

TNBC has the highest mutational burden among breast cancer subtypes, generating more neoantigens and eliciting greater baseline immune infiltration. Key immunosenescence features specifically documented in TNBC include the ISG+ senescent CD8+ T cell population that mediates anti-PD-1 resistance (5), diminished IFN signaling with age correlating with ICI outcomes (6), age-related declines in TIL density being most pronounced (41), and M2 macrophage and exhausted T cell co-enrichment (12). However, whether these findings are specific to TNBC or simply more readily detected due to higher baseline immune activity is unclear, as comparable studies in other subtypes are lacking.

#### 4.2.2 HER2-positive breast cancer

HER2+ tumors exhibit intermediate immunogenicity and are uniquely treated with HER2-targeted antibodies (trastuzumab, pertuzumab) that engage antibody-dependent cellular cytotoxicity (ADCC) mediated by NK cells. Age-related declines in NK cell cytotoxic function and CD16 (FcγRIII) expression may compromise ADCC-mediated tumor clearance—a subtype-specific vulnerability to NK cell immunosenescence. The impact of antibody-drug conjugates (T-DM1, T-DXd) on immune senescence in the TME has not been studied.

#### 4.2.3 Hormone receptor-positive breast cancer

HR+ tumors typically harbor lower mutational burdens, fewer TILs, and a less immunogenic TME at baseline. Immunosenescence in HR+ disease may manifest more subtly—as alterations in Treg/effector T cell balance, changes in TLS formation, or shifts in macrophage polarization—rather than as overt TIL depletion. The paradoxical association of higher Tregs with improved survival in young HR+ patients (42) suggests that the relationship between immune senescence and clinical outcomes may be qualitatively different in HR+ compared with TNBC disease.

The subtype specificity of immunosenescence has practical consequences: age-adjusted TIL thresholds and senescence markers may need to be calibrated separately for each subtype, and senotherapeutic strategies may need to be tailored accordingly—for example, NAD+ repletion (NMN) in TNBC where ISG+ T cell senescence is prominent, senolytic CAF targeting in HR+ disease, and NK cell rejuvenation in HER2+ disease to preserve ADCC. These subtype-specific hypotheses require prospective testing.

### 4.3 Hormonal signaling, menopausal status, and immunosenescence

Hormonal signaling—particularly through estrogen and progesterone—exerts profound immunomodulatory effects, and the dramatic hormonal changes associated with menopause have significant implications for tumor immunosenescence in breast cancer.

Estrogen and immune function. Estrogen promotes thymic cellularity and thymopoiesis, enhances B cell lymphopoiesis and antibody production, promotes Th1 polarization and IFN-γ production, increases CD8+ T cell cytotoxic activity, and stimulates NK cell function. Pre-menopausal estrogen levels may confer a degree of protection against age-related immune decline; the loss of estrogen signaling at menopause removes this protective effect, potentially accelerating immunosenescence in post-menopausal women.

Menopause and the breast TME. Menopause is associated with increased adipose tissue content and altered adipokine secretion (elevated leptin, reduced adiponectin), shifts in the breast-resident microbiome, and increased systemic inflammaging markers. These changes coincide temporally with the peak incidence of breast cancer diagnosis (median age ~62 years), creating a biological context in which menopause-associated immune changes, chronological immunosenescence, and tumor-driven immune suppression converge.

Pre- versus post-menopausal immunosenescence signatures. Pre-menopausal women develop breast cancer against a background of relatively preserved immune function, and their tumors may be dominated by tumor-driven immune suppression rather than host-driven immunosenescence. Post-menopausal women face the combined effects of chronological immune aging, menopause-related immune changes, and tumor-driven immune suppression—a triple insult that may produce a more profound immunosenescence phenotype. Dedicated studies comparing immune parameters in pre- versus post-menopausal breast cancer patients, controlling for chronological age and tumor subtype, are needed.

Endocrine therapy and immunosenescence. Aromatase inhibitors (AIs) and tamoxifen, the mainstays of adjuvant endocrine therapy for HR+ breast cancer, may themselves influence immune senescence. AIs lower circulating estrogen further, potentially exacerbating the immune effects of estrogen loss. Tamoxifen, as a SERM, has tissue-specific effects that may differ from those of AIs. The widespread use of endocrine therapy for 5–10 years creates a large population in whom drug-induced hormonal modulation may interact with immunosenescence over an extended period—an important but understudied area.

### 4.4 Immunosenescence across progressive stages of breast cancer

The immune landscape of breast cancer evolves dynamically as the disease progresses from pre-invasive lesions to invasive carcinoma to metastatic disease. Immunosenescence likely modifies this trajectory at each stage, though direct evidence is sparse.

Pre-invasive to invasive transition. Age-related impairments in immune surveillance—reduced naive T cell output, restricted TCR repertoire, diminished NK cell function—may increase the probability that DCIS lesions escape immune control and progress to invasive carcinoma. Studies comparing the immune microenvironment of DCIS lesions from younger versus older women using multiplexed imaging or single-cell approaches could test this hypothesis.

Primary invasive disease. Key stage-specific considerations include TIL density and composition differences between stage I, II, and III disease, with age potentially modifying these patterns; the accumulation of senescent T cells and CAFs that may increase with tumor size and disease duration; and lymph node metastasis-associated T cell dysfunction that may be compounded by age (13).

Metastatic disease. Age-related immune dysfunction may facilitate both survival of circulating tumor cells (reduced NK cytotoxicity) and establishment of the pre-metastatic niche (myeloid skewing, systemic inflammation). The bone marrow, a common site of breast cancer metastasis, is also the primary site of age-related hematopoietic changes; whether age-related bone marrow remodeling creates a more permissive niche for metastatic colonization is an important question. Direct comparisons of the immune microenvironment in primary versus metastatic tumors stratified by age would be informative but are logistically challenging.

### 4.5 BRCA mutations and susceptibility to immunosenescence

Germline mutations in BRCA1 and BRCA2 are the most established genetic risk factors for breast cancer. Several lines of reasoning suggest that BRCA mutation carriers may exhibit altered susceptibility to immunosenescence, though direct evidence is extremely limited.

BRCA-mutant tumors are characterized by homologous recombination deficiency (HRD), which generates higher levels of cytosolic DNA and activates the cGAS-STING pathway, leading to increased type I IFN production and enhanced T cell priming. However, as demonstrated by Fu and colleagues, sustained type I IFN signaling can paradoxically drive CD8+ T cell senescence through NAD+ depletion (5), suggesting that BRCA-mutant tumors with chronic STING activation may be at risk for IFN-driven T cell senescence. Additional considerations include: (i) BRCA mutations may also affect the DNA damage response in immune cells, potentially accelerating immune cell senescence following genotoxic therapy; (ii) risk-reducing salpingo-oophorectomy in BRCA carriers may alter immune parameters through surgical menopause; (iii) PARP inhibitor therapy may induce senescence in both tumor and immune cells; and (iv) the younger median age at breast cancer diagnosis for BRCA1 carriers (~40–45 years versus ~62 years for sporadic cases) means BRCA-associated tumors arise in a chronologically younger immune environment, which may partially offset BRCA-related susceptibility to immunosenescence. Systematic comparison of immunosenescence markers between BRCA-mutant and sporadic breast cancer patients, controlling for age and tumor subtype, represents an important research opportunity.

### 4.6 Aging and the evolution of the TME from pre-neoplasia to metastasis

The TME immune composition at each disease stage—atypical hyperplasia, *in situ* carcinoma, invasive carcinoma, locoregional recurrence, and distant metastasis—reflects the interplay between tumor-intrinsic factors and host-intrinsic factors (immune competence, inflammaging, thymic function, bone marrow reserve). In young hosts with intact immune systems, tumor progression is constrained by effective immune surveillance. In aged hosts with impaired immune surveillance, the selection pressure imposed by the immune system is weaker, potentially permitting the outgrowth of more immunogenic tumor clones that would be eliminated in a younger host.

Key knowledge gaps include: (i) whether the rate of immune cell infiltration decline with age is constant across disease stages or accelerates at specific transitions (e.g., DCIS to invasive carcinoma); (ii) whether the relative proportions of exhausted versus senescent T cells shift with disease progression and whether age modifies this shift; (iii) whether the spatial organization of immune cells within the TME—immune-excluded, immune-desert, or immune-inflamed phenotypes—varies with age and disease stage; and (iv) whether age-associated changes in the pre-metastatic niche precede or follow metastatic dissemination. Addressing these questions will require longitudinal sampling of the TME across disease stages in age-stratified patient cohorts, increasingly feasible with technologies such as circulating tumor DNA (ctDNA) immune profiling and serial biopsy analysis.

### 4.7 Single-cell and spatial profiling technologies: advancing our understanding of immunosenescence

Recent advances in single-cell and spatial profiling technologies have substantially refined our understanding of immune cell heterogeneity, stromal-immune interactions, and tissue organization within the breast TME. These technologies offer unprecedented opportunities to dissect immunosenescence at high resolution.

Single-cell RNA sequencing (scRNA-seq) has enabled the identification of transcriptionally distinct senescent and exhausted T cell subsets, including the ISG+CD8+ T cell population (5) and co-exhaustion states defined by PD-1, TIM-3, and LAG-3 co-expression (12). Single-cell ATAC-seq (scATAC-seq) maps chromatin accessibility and can identify the regulatory programs underlying T cell exhaustion (TOX-driven) versus senescence (DDR/p53/p16-driven). CITE-seq and single-cell proteomics enable simultaneous measurement of transcriptomes and surface protein expression, facilitating the development of flow cytometry panels that operationally distinguish senescent from exhausted T cells. Spatial transcriptomics (10x Visium, MERFISH, Xenium) preserves the spatial context of gene expression, enabling analysis of how senescent cells are organized within the TME. Multiplexed imaging (CyCIF, MIBI-TOF, CODEX, IMC) allows simultaneous detection of 30-100+ protein markers on a single tissue section, ideally suited for studying the spatial organization of immunosenescence in archival breast cancer specimens.

Challenges include: high cost and technical complexity limiting studies to small, single-institution cohorts; fresh tissue requirements for some assays precluding analysis of archival specimens with known clinical outcomes; lack of standardized computational pipelines for senescence cell identification in single-cell datasets; and absence of consensus markers for defining senescent cells. Despite these challenges, the systematic application of single-cell and spatial technologies to age-stratified breast cancer cohorts represents a high-priority research direction with the potential to transform our understanding of immunosenescence at the tissue level.

### 4.8 Biomarker standardization and clinical implementation challenges

While several candidate biomarkers of immunosenescence have been identified, their translation into clinical practice faces substantial obstacles that warrant explicit discussion.

Lack of consensus definitions. There is currently no consensus on which markers define a cell as ‘senescent’ versus ‘exhausted’ versus ‘dysfunctional’ in human tumor specimens. SA-β-gal activity is the most commonly used senescence marker but requires fresh or frozen tissue and has limited specificity. p16INK4a immunohistochemistry is more practical for FFPE specimens but lacks standardized scoring criteria. The absence of a validated, field-wide consensus marker panel for immunosenescence is arguably the single greatest obstacle to clinical translation.

Inter-laboratory variability. Even for well-established biomarkers such as TIL density, inter-observer and inter-laboratory variability remain significant. For senescence-specific markers, variability is even greater due to the lack of standardized protocols, antibodies, and scoring systems.

Validation across patient cohorts. Most immunosenescence biomarker studies in breast cancer have been conducted in single-institution cohorts with limited sample sizes, varying definitions of ‘older’ patients, and inconsistent adjustment for potential confounders. Independent validation in large, multi-institutional, age-diverse cohorts with standardized biospecimen collection and biomarker assessment protocols has not been performed for any immunosenescence biomarker in breast cancer.

Dynamic versus static assessment. A single biopsy captures the immune state at one time point, but immunosenescence is a dynamic process that evolves over time and in response to therapy. Serial biomarker assessment—through liquid biopsies (circulating senescent T cells, SASP factors, cell-free DNA with senescence-associated methylation patterns) or repeat tumor biopsies—may provide more clinically useful information than single time-point measurements but adds cost, invasiveness, and logistical complexity. **Table 2** summarizes the key immunosenescence biomarkers currently under investigation in breast cancer, their detection methods, associations with age, and clinical prognostic and predictive significance.

**Table 2:** Immunosenescence biomarkers and clinical evidence in breast cancer.

Biomarker	Detection method	Association with age	Prognostic significance	Predictive value for ICI efficacy
TIL density	H&E staining; IHC for CD3/CD8	Lower TIL density in older (>60 yr) vs younger (≤45 yr) TNBC patients; within young HR^+^ cohort (≤40 yr), higher immune infiltration paradoxically associated with increasing age (41, 42)	Higher TIL density → improved pCR after neoadjuvant chemotherapy and better OS, especially in TNBC and HER2-enriched subtypes (40); age-stratified analysis essential	Higher TILs generally favorable, but age-dependent immunosenescence may confound predictive accuracy
p16^INK4a	IHC; qPCR in peripheral blood T cells	Expression in peripheral T cells increases exponentially with chronological age; correlates with CD8^+^CD28^-^ senescent T cell accumulation (9)	Higher p16^INK4a in TILs associated with advanced disease and poorer neoadjuvant chemotherapy response (46); requires prospective validation	Not yet established as an independent ICI predictor
IFN signaling signatures	RNA-seq; NanoString gene expression panels	Progressively attenuated with age in both murine TNBC models and human TNBC patients (6); diminished IFN-γ and type I IFN gene signatures in older patients	Attenuated IFN signaling independently associated with worse ICI outcome in TNBC (6); may require age-adjusted thresholds	STING agonist-mediated restoration of IFN signaling rescued ICI responsiveness in aged mice (6)
ISG^+^CD8^+^ T cell frequency	scRNA-seq; flow cytometry (ISG markers); derived gene signature	Enriched in early-stage TNBC resistant to anti-PD-1; driven by monocyte-derived type I IFN via IRF9–PARP–NAD^+^ axis (5)	Predicts anti-PD-1 resistance in early TNBC; identifies patients who may benefit from NAD^+^ repletion (e.g., NMN) rather than ICI alone (5)	Direct predictive biomarker for anti-PD-1 failure; NAD^+^ repletion restores ICI responsiveness
Treg/PD-L1 (HR^+^ young patients)	Multiplex IF; IHC for FOXP3, PD-L1	Higher stromal Treg and PD-L1 expression paradoxically associated with improved iBCFS and DDFS in young (≤40 yr) HR^+^/HER2^-^ patients (42)	Challenges conventional view that Tregs are uniformly detrimental; age and subtype context critical for interpretation (42, 43)	Higher PD-L1 may still indicate ICI benefit, but Treg prognostic complexity demands nuanced interpretation
Peripheral blood immune profiles	Flow cytometry; mass cytometry (CyTOF)	Age-related shifts in CD4:CD8 ratio, naive-to-memory T cell ratio, MDSC frequency, and reduced TCR repertoire diversity (4)	Correlated with cancer prognosis and treatment outcomes; integration with tumor tissue biomarkers recommended	May reflect systemic host immune competence; predictive value under investigation

H&E, hematoxylin and eosin; IHC, immunohistochemistry; IF, immunofluorescence; pCR, pathological complete response; OS, overall survival; iBCFS, invasive breast cancer-free survival; DDFS, distant disease-free survival; scRNA-seq, single-cell RNA sequencing.

Integration with clinical decision-making. Even with analytically and clinically validated biomarkers, their integration into treatment algorithms requires demonstrating that biomarker-guided therapy improves patient outcomes. Biomarker-driven clinical trials specifically designed to test immunosenescence-stratified therapeutic strategies have not yet been initiated in breast cancer.

Practical considerations. For a biomarker to be clinically adopted, it must be measurable in routinely available specimens (FFPE tissue, peripheral blood) using assays that are affordable, reproducible, and compatible with standard clinical laboratory workflows. Many of the most informative senescence assays (single-cell sequencing, multiplexed imaging, telomere length measurement by Flow-FISH) currently fall outside these practical constraints.

### 4.9 Impact of immunosenescence on immune checkpoint inhibitor efficacy

Immune checkpoint inhibitors targeting the PD-1/PD-L1 and CTLA-4 axes have demonstrated clinical activity in breast cancer, with the most compelling benefit observed in TNBC. The KEYNOTE-522 trial established pembrolizumab (anti-PD-1) combined with chemotherapy as a new standard of care for early-stage TNBC, and the IMpassion130 trial demonstrated the benefit of atezolizumab (anti-PD-L1) plus nab-paclitaxel in PD-L1-positive metastatic TNBC (44). However, not all patients benefit, and durable responses remain infrequent in the metastatic setting. Emerging evidence indicates that immunosenescence may be a significant contributor to both primary and acquired resistance to ICIs in breast cancer, though most data derive from preclinical models and retrospective subgroup analyses rather than from trials designed to evaluate age as a primary variable.

Sceneay and colleagues provided one of the most direct demonstrations that age-associated immune dysfunction limits ICI efficacy (6). In murine TNBC models, aged mice exhibited significantly reduced responses to combined anti-CTLA-4 and anti-PD-L1 therapy compared with young mice. Mechanistically, both aged mice and older human TNBC patients exhibited diminished IFN signaling and impaired antigen presentation in the tumor microenvironment. Gene expression analysis of pretreatment tumor specimens from TNBC patients treated with ICIs revealed that IFN-related gene signatures were progressively attenuated with increasing patient age. Critically, innate immune priming with a STING agonist restored ICI responsiveness in aged mice, providing proof-of-principle that age-related immune defects can be pharmacologically overcome (6). However, the clinical component of this study was limited to a small, retrospectively analyzed cohort (n = 38 patients), and the finding that IFN signatures decline linearly with age in TNBC has not been independently replicated in larger, multi-institutional cohorts. Furthermore, it remains unclear whether the age-ICI relationship observed in this study is specific to TNBC or generalizable across breast cancer subtypes.

The discovery of the ISG^+^CD8^+^ T cell subset by Fu and colleagues provides complementary mechanistic insight (5). In early-stage TNBC, the abundance of ISG^+^ senescent CD8^+^ T cells predicted resistance to anti-PD-1 therapy. These cells, driven into senescence by monocyte-derived type I IFN, exhibited metabolic exhaustion characterized by NAD^+^ depletion and were no longer capable of responding to PD-1 blockade. Importantly, supplementation with nicotinamide mononucleotide (NMN), an NAD^+^ precursor, restored the metabolic fitness and cytotoxic function of senescent CD8^+^ T cells and enhanced anti-PD-1 efficacy in patient-derived organoid co-culture and murine models (5). This work identifies a specific mechanism through which innate immune signaling paradoxically drives T cell senescence and ICI resistance and nominates NAD^+^ repletion as a potential therapeutic intervention.

The broader literature on aging and ICI efficacy in solid tumors supports the concept that older patients may derive less benefit from checkpoint blockade. A comprehensive review by Elias and colleagues documented that age-related changes in T cell repertoire diversity, CD28 expression, and co-inhibitory receptor expression influence ICI outcomes, though the directionality varies by tumor type and the specific checkpoint pathway targeted (45). In breast cancer, the available clinical evidence is suggestive but inconclusive: suggests that age should be incorporated into predictive models of ICI benefit and that age-adapted combination strategies may be necessary to achieve optimal outcomes in older patients. Critically, most pivotal ICI trials in breast cancer have under-enrolled patients over 70 years of age, limiting the evidence base for this demographic that constitutes a large proportion of real-world breast cancer patients.

### 4.10 Immunosenescence markers as prognostic and predictive biomarkers

The identification of robust biomarkers that capture the functional state of the immune system—beyond simple enumeration of immune cell subsets—is a critical priority for precision immuno-oncology. Several senescence-associated biomarkers have shown promise in breast cancer.

p16^INK4a. The cyclin-dependent kinase inhibitor p16^INK4a is one of the most widely used markers of cellular senescence and aging. In peripheral blood T cells, p16^INK4a expression increases exponentially with chronological age and correlates with the accumulation of senescent CD8^+^CD28^-^ T cells (9). In breast cancer patients, higher p16^INK4a expression in tumor-infiltrating T cells has been associated with more advanced disease and poorer response to neoadjuvant chemotherapy, though prospective validation in larger cohorts is needed (46).

IFN Signaling Signatures. The IFN-γ and type I IFN gene expression signatures have been extensively evaluated as predictive biomarkers for ICI response. The study by Sceneay and colleagues demonstrated that an IFN-related gene signature was progressively attenuated with age in TNBC patients and was independently associated with ICI outcome, suggesting that these signatures may need age-adjusted thresholds for optimal clinical utility (6).

ISG^+^CD8^+^ T Cell Frequency. The frequency of ISG^+^ senescent CD8^+^ T cells, identified by single-cell analysis or a derived gene signature, predicted anti-PD-1 resistance in early TNBC (5). If validated in independent cohorts, this biomarker could identify patients unlikely to benefit from PD-1 blockade alone, who might be candidates for combination strategies that restore T cell metabolic fitness (**Table 2**).

TIL Subtype Composition. The composition of TILs—rather than total density alone—may provide more nuanced prognostic information, particularly in young patients with HR^+^ disease. The finding that higher Treg and PD-L1 positivity was associated with improved survival in young HR^+^ breast cancer patients challenges simplified models of immune suppression and highlights the need for age-stratified biomarker analyses (42, 43).

Circulating Immune Cell Profiles. Age-related changes in peripheral blood immune cell composition, including the CD4:CD8 ratio, naive-to-memory T cell ratio, and MDSC frequency, have been correlated with cancer prognosis and treatment outcomes (4). The integration of peripheral blood immunosenescence markers with tumor tissue biomarkers may enable a more comprehensive assessment of host immune competence and its impact on therapeutic response. **Table 3** provides a structured overview of these emerging strategies, their mechanisms, stages of development, and key challenges.

**Table 3:** Emerging therapeutic strategies targeting tumor immunosenescence in breast cancer.

Therapeutic strategy	Representative agents	Mechanism of action	Preclinical/clinical evidence	Research stage	Key challenges
Senolytics: Navitoclax (ABT-263)	BCL-2/BCL-XL/BCL-W inhibitor	Eliminates senescent tumor cells post-TIS; effective in combination with radiation + PARP inhibition (“one-two punch” strategy)	Significantly delayed tumor regrowth in TNBC models after radiation- or chemotherapy-induced senescence (38)	Preclinical	Dose-limiting thrombocytopenia (BCL-XL inhibition in platelets)
Senolytics: Venetoclax	BCL-2-selective inhibitor	Cell line-dependent senolytic activity; effective against E0771 but not 4T1 senescent cells; enhanced efficacy with radiotherapy + talazoparib (39, 49)	Eliminated therapy-induced senescent TNBC cells in immunocompetent mouse models (39)	Preclinical	Variable sensitivity across breast cancer subtypes; biomarker-guided patient selection needed
Senolytics: Dasatinib + Quercetin	Multi-kinase inhibitor + flavonoid (PI3K/AKT/BCL-2)	First senolytic regimen to enter clinical trials; reduces senescent cell burden and SASP-mediated tissue dysfunction	Improved physical function and reduced circulating SASP factors in clinical trials; tumor-specific efficacy data limited (47, 48)	Phase I/II clinical trials	Long-term safety; tumor-specific efficacy not yet demonstrated
Senolytics: Fisetin	Naturally occurring flavonoid	PI3K/AKT and BCL-2 pathway inhibition; both senolytic and senomorphic properties	Reduces age-related pathology in murine models; favorable safety profile (47, 48)	Phase I/II trials (frailty, CKD, cancer survivorship)	Oral bioavailability; optimal dosing schedule not established
Senomorphics: JAK/STAT inhibitors	Ruxolitinib, other JAK1/2 inhibitors	Suppress JAK2/STAT3-driven SASP expression (IL-6, IL-8, CCL2)	Reduce SASP factor secretion and alleviate age-related inflammation in preclinical models (47, 51)	Preclinical	Broad immunosuppression concerns
Senomorphics: NF-κB inhibitors	IKK inhibitors, IκBα super-repressor	Inhibit master NF-κB transcriptional regulator of SASP	Suppress SASP across multiple senescence models (31)	Preclinical	Pleiotropic NF-κB functions in normal immunity; need cell-type-specific delivery
Senomorphics: mTOR inhibitors	Rapamycin, rapalogs	Suppress SASP translation via mTOR/S6K pathway; activate autophagy	Extend lifespan and healthspan in model organisms; reduce SASP-mediated tumor promotion (48)	Preclinical/Repurposing	Metabolic side effects with chronic use
Senomorphics: Metformin	AMPK activator	Senomorphic SASP suppression via AMPK-mediated NF-κB inhibition	Associated with reduced cancer incidence in epidemiological studies; evaluated as breast cancer adjuvant therapy (47)	Phase III adjuvant trials	Modest effect size; optimal patient population unclear
Metabolic reprogramming	NMN (NAD^+^ precursor), MitoQ, MitoTEMPO	NMN: restores NAD^+^ levels and rescues mitochondrial respiration in senescent ISG^+^CD8^+^ T cells; MitoQ: mitochondrial-targeted antioxidant	NMN restored anti-PD-1 efficacy in patient-derived organoid co-culture and murine TNBC models (5)	Preclinical (NMN); early preclinical (MitoQ)	Long-term NAD^+^ repletion safety; tumor-promoting effects of NAD^+^ in some contexts
STING agonists	cGAMP, ADU-S100, non-nucleotide agonists	Activate cytosolic DNA sensing → type I IFN production → DC maturation and T cell priming	Restored IFN signaling and ICI responsiveness in aged TNBC mice (6)	Phase I/II trials (solid tumors)	Excessive IFN signaling may paradoxically drive CD8^+^ T cell senescence; narrow therapeutic window (5)
ICB optimization	Anti-PD-1 + anti-CTLA-4, anti-TIGIT, anti-LAG-3; IL-15 superagonists	Multi-checkpoint blockade overcomes higher T cell activation threshold in aged hosts; pre-chemotherapy ICI preserves thymic function	Combined anti-PD-1 + anti-CTLA-4 extended time to relapse in doxorubicin-treated breast tumor models (34)	Preclinical/rational design	Under-enrollment of patients >70 yr in ICI trials; lack of age-specific trial designs
Adoptive cell therapy	CAR T cells (senescent cell-targeted: anti-uPAR, anti-DPP4); TCR-engineered T cells	Bypass age-related endogenous immune defects; selection of less differentiated T cell subsets; knockout of senescence genes (p16, PD-1)	CAR T cells targeting senescent cell surface markers under development (48, 53)	Preclinical	Antigen heterogeneity in solid tumors; CAR T cell dysfunction in immunosuppressive TIME
Combination strategies (S+T+I)	Senescence induction (radiation, PARPi) → Senolytic clearance (navitoclax) → Immune activation (ICI); STING agonist + ICI	Stepwise: immune priming → ICI amplification → intermittent senolytic dosing (“hit-and-run”)	Superior tumor control with combined senolytic + dual ICI vs either approach alone (34); multi-step S+T+I regimen demonstrated feasibility (38, 39)	Preclinical/early clinical	Complex optimization of agent choice, dose, schedule, and sequence; need for dynamic immune monitoring biomarkers
Microbiota modulation [RED: NEW]	FMT, probiotics (Bifidobacterium, Akkermansia), dietary interventions, antibiotics	Restore age-depleted beneficial commensals; microbial metabolites (SCFAs) support immune homeostasis; modulate systemic inflammation and estrogen metabolism	FMT from young to aged mice reverses some age-related immune phenotypes in preclinical models; no breast cancer-specific immunosenescence trials	Preclinical to early clinical	Low breast tissue microbial biomass (contamination risk); correlative human data; functional causality not established; unregulated probiotic market; context-dependent microbial effects

TIS, therapy-induced senescence; CKD, chronic kidney disease; MitoQ, mitoquinone mesylate; ICB, immune checkpoint blockade; S+T+I, senescence induction + targeted senolysis + immunotherapy.

## 5 Emerging therapeutic interventions

A range of therapeutic strategies targeting tumor immunosenescence are under investigation. However, it is essential to emphasize that the vast majority of these strategies remain at preclinical or early clinical stages of development. While the preclinical data are mechanistically compelling, only a small subset of agents have entered clinical trials, and none have demonstrated tumor-specific efficacy in randomized trials in breast cancer patients. The translational recommendations that follow should be interpreted as hypotheses requiring rigorous clinical evaluation rather than as established therapeutic paradigms.

### 5.1 Senolytic therapies

Senolytics are pharmacological agents that selectively eliminate senescent cells by exploiting their unique survival dependencies, particularly their reliance on anti-apoptotic networks. Senescent cells upregulate pro-survival pathways including the BCL-2 family, PI3K/AKT, and HSP90, which protect them from their own pro-apoptotic SASP milieu. By inhibiting these pathways, senolytics induce apoptosis specifically in senescent cells while sparing non-senescent cells (47, 48). This mechanism has been validated across multiple tissue types in preclinical models, but the selectivity, long-term safety, and clinical efficacy of senolytic therapy in cancer patients—particularly older patients with pre-existing immunosenescence—have not been established.

Navitoclax (ABT-263). Navitoclax, an orally bioavailable inhibitor of BCL-2, BCL-XL, and BCL-W, has been the most extensively studied senolytic in breast cancer models. In TNBC, navitoclax administered after radiation- or chemotherapy-induced senescence effectively eliminated senescent tumor cells and significantly delayed tumor regrowth *in vivo* (38). The combination of radiation with PARP inhibition enhanced senescence induction and sensitized TNBC cells to navitoclax-mediated senolysis, demonstrating that the “one-two punch” strategy can be optimized through rational drug sequencing (38). However, the clinical development of navitoclax as a senolytic is complicated by dose-limiting thrombocytopenia resulting from BCL-XL inhibition in platelets, highlighting the need for BCL-2-selective agents or intermittent dosing schedules. and whether intermittent “hit-and-run” dosing can achieve adequate senolytic efficacy while maintaining a tolerable safety profile in older breast cancer patients is unknown.

Venetoclax. Venetoclax, a BCL-2-selective inhibitor with an improved safety profile relative to navitoclax, has shown cell line–dependent senolytic activity in TNBC models. While venetoclax was effective against therapy-induced senescent E0771 cells, it had minimal activity against senescent 4T1 cells, indicating that biomarkers of senolytic susceptibility will be essential for patient selection (49). The combination of venetoclax with radiotherapy and talazoparib produced encouraging results in immunocompetent mouse models (39). This cell line-dependent variability underscores a broader challenge: senescent cells from different tumors may exhibit different anti-apoptotic dependencies, and a single senolytic agent may not eliminate all senescent populations.

Dasatinib plus Quercetin. The combination of dasatinib (a tyrosine kinase inhibitor) and quercetin (a flavonoid that inhibits PI3K/AKT and BCL-2 family proteins) was the first senolytic regimen to demonstrate efficacy in preclinical models and has subsequently entered clinical trials. In mouse models, intermittent administration of dasatinib plus quercetin reduced the burden of senescent cells, alleviated age-related physical dysfunction, and extended healthspan (48). In cancer models, this combination has shown activity in eliminating therapy-induced senescent cells and reducing SASP-mediated tumor-promoting effects. Clinical trials evaluating dasatinib plus quercetin in cancer survivors and patients with age-related conditions have reported improvements in physical function and reductions in circulating SASP factors, though tumor-specific efficacy data remain limited (47). Importantly, these trials were not designed to evaluate cancer outcomes, and whether dasatinib plus quercetin can delay recurrence, prevent metastasis, or improve survival in breast cancer patients is not known.

Fisetin. Fisetin, a naturally occurring flavonoid with senolytic and senomorphic properties, has attracted considerable interest due to its favorable safety profile. Fisetin eliminates senescent cells in multiple tissues and reduces age-related pathology in murine models (48). Its senolytic activity is mediated through the inhibition of the PI3K/AKT and BCL-2 pathways. Clinical trials evaluating fisetin in the context of frailty, chronic kidney disease, and cancer survivorship are ongoing (47). As with dasatinib plus quercetin, cancer-specific efficacy data are not yet available.

Emerging Senolytics. Beyond these first-generation agents, several next-generation senolytic strategies are under investigation. Ferroptosis induction has been identified as a novel senolytic approach, exploiting the observation that therapy-induced senescent cells accumulate lysosomal ferrous iron, rendering them selectively vulnerable to ferroptosis (50). CAR T cells engineered to recognize senescent cell surface markers (e.g., uPAR, DPP4) are being developed as a targeted senolytic strategy. Prodrugs activated by the lysosomal β-galactosidase activity characteristic of senescent cells offer another approach for selective senescent cell elimination (48). These approaches are at very early stages of development and their feasibility in breast cancer patients has not been demonstrated.

### 5.2 Senomorphic strategies targeting SASP

Senomorphics are agents that suppress the SASP without necessarily eliminating senescent cells. This approach may be preferable in contexts where the tumor-suppressive function of senescence (permanent cell cycle arrest) is desired while the tumor-promoting effects of the SASP need to be contained (47). However, a theoretical concern is that long-term senomorphic therapy may allow the accumulation of senescent cells that could, upon drug discontinuation, resume SASP production and drive disease progression.

JAK/STAT Inhibitors. The JAK2/STAT3 signaling axis is a central regulator of SASP expression, and pharmacological inhibition of this pathway suppresses the production of multiple SASP factors including IL-6, IL-8, and CCL2. In breast cancer, metochalcone was shown to induce SASP via the JAK2/STAT3 pathway, suggesting that JAK inhibition may be a relevant senomorphic strategy (51). Ruxolitinib and other JAK1/2 inhibitors have been shown to reduce SASP factor secretion from senescent cells and alleviate age-related inflammation in preclinical models (47).

NF-κB Inhibitors. NF-κB is the master transcriptional regulator of the SASP. Inhibitors of the IKK complex and other components of the NF-κB pathway suppress SASP expression across multiple senescence models. However, the pleiotropic functions of NF-κB in normal immune responses necessitate cell-type-specific or locally delivered inhibitors to avoid systemic immunosuppression (31).

mTOR Inhibitors. The mTOR pathway is activated in senescent cells and contributes to SASP production through enhanced mRNA translation of SASP factors and activation of NF-κB. Rapamycin and its analogs (rapalogs) suppress the SASP and have been shown to extend lifespan and healthspan in model organisms (48). In cancer models, mTOR inhibition reduces SASP-mediated tumor-promoting effects and may synergize with senolytics.

Metformin. The widely used anti-diabetic drug metformin has senomorphic properties, suppressing SASP factor secretion from senescent cells through AMPK-mediated inhibition of NF-κB. Epidemiological studies have associated metformin use with reduced cancer incidence and improved cancer outcomes, and several clinical trials are evaluating metformin as an adjuvant therapy in breast cancer (47).

Glucocorticoids. Corticosteroids are potent suppressors of inflammatory SASP factors including IL-6 and IL-8. However, their broad immunosuppressive effects and metabolic toxicity limit their utility as chronic senomorphic agents. Local or intermittent glucocorticoid delivery approaches may mitigate these concerns.

### 5.3 Metabolic reprogramming of senescent immune cells

The metabolic dimension of T cell senescence has opened new therapeutic opportunities. The discovery that senescent ISG^+^CD8^+^ T cells in TNBC suffer from NAD^+^ depletion due to chronic type I IFN signaling identified a specific metabolic vulnerability that can be therapeutically targeted (5). Supplementation with NMN, an NAD^+^ precursor, restored the metabolic capacity, effector function, and anti-PD-1 responsiveness of these senescent T cells in preclinical models. This approach represents a fundamentally new therapeutic concept: rather than eliminating senescent cells (senolysis) or suppressing their secretome (senomorphism), metabolic rescue aims to reverse the senescent phenotype and restore functional competence. However, several critical issues must be addressed before clinical translation: (i) the long-term safety of NAD+ precursor supplementation in cancer patients has not been established, and NAD+ can theoretically fuel both anti-tumor immune cells and tumor cells; (ii) the optimal dose, formulation, and schedule of NMN or other NAD+ precursors in humans are not defined; (iii) which patients are most likely to benefit is unknown; and (iv) whether metabolic rescue produces durable immune restoration or merely transient improvement has not been determined.

Beyond NAD^+^ repletion, other metabolic interventions are being explored. Mitochondrial dysfunction with elevated ROS production is a hallmark of senescent T cells, and mitochondrial-targeted antioxidants (e.g., MitoQ, MitoTEMPO) have shown promise in restoring T cell function in aged mice. Modulation of fatty acid oxidation, glycolysis, and mitochondrial biogenesis through pharmacological agents (e.g., AMPK activators, PPAR agonists) represents additional strategies for rejuvenating aged T cells (52).

### 5.4 STING agonists and innate immune priming

The observation that age-associated impairment of IFN signaling in TNBC could be rescued by STING agonist treatment provided an important proof-of-principle (6). STING (stimulator of interferon genes) is a key sensor of cytosolic DNA that activates type I IFN production and promotes DC maturation and T cell priming. Cyclic dinucleotide STING agonists and next-generation non-nucleotide agonists have entered clinical trials in solid tumors, including breast cancer. By restoring IFN signaling and enhancing antigen presentation, STING agonists may help overcome the age-related deficit in innate immune activation that limits ICI efficacy in older patients. However, the therapeutic window for STING agonists in the context of immunosenescence—delivering sufficient STING activation to prime anti-tumor immunity without triggering the IRF9-PARP-NAD+ depletion axis that drives T cell senescence—will require careful definition, potentially through optimized dosing schedules, local rather than systemic delivery, or combination with NAD+ repletion to prevent senescence induction.

However, the relationship between STING activation and immunosenescence is complex. While acute STING activation promotes anti-tumor immunity, the recent finding that monocyte-derived type I IFN drives CD8^+^ T cell senescence through NAD^+^ depletion (5) cautions that sustained or excessive IFN signaling may paradoxically promote immune dysfunction. The therapeutic window for STING agonists in the context of immunosenescence will require careful definition.

### 5.5 Optimizing immune checkpoint blockade

Several strategies are being explored to optimize ICI efficacy in the context of immunosenescence. First, combination checkpoint blockade targeting multiple co-inhibitory pathways (e.g., anti-PD-1 plus anti-CTLA-4, anti-PD-1 plus anti-TIGIT, anti-PD-1 plus anti-LAG-3) may overcome the higher threshold for T cell activation in aged hosts. The observation that a combination of anti-PD-1 and anti-CTLA-4 extended time to relapse in doxorubicin-treated breast tumors harboring heterogeneous senescent populations supports this approach (34). These remain largely at the rational design stage and have not been tested in age-stratified randomized trials. Clinical trials specifically designed to evaluate ICI-based regimens in older breast cancer patients are urgently needed, as the pivotal trials establishing ICI efficacy in breast cancer under-enrolled patients over 70 years of age.

Second, agents that reverse T cell exhaustion and senescence—such as NMN for metabolic rescue (5) or IL-15 superagonists that promote NK and CD8^+^ T cell proliferation—may enhance ICI responsiveness when used as priming or concurrent therapy. Third, the timing and sequencing of ICIs relative to chemotherapy and radiotherapy are likely to be critical in aged hosts. Chemotherapy-induced acute thymic involution and lymphodepletion may be particularly detrimental in older patients with pre-existing immunosenescence, suggesting that ICI administration before or concurrent with, rather than after, chemotherapy may be more effective. Clinical trials specifically designed to evaluate ICI-based regimens in older breast cancer patients are urgently needed.

### 5.6 Adoptive cell therapy and TCR engineering

For patients with advanced immunosenescence who lack functional endogenous anti-tumor immunity, adoptive cell therapy (ACT) offers a strategy to bypass age-related immune defects altogether. CAR T cell therapy has achieved remarkable success in hematologic malignancies, but its application to solid tumors, including breast cancer, faces significant challenges including antigen heterogeneity, immunosuppressive tumor microenvironments, and T cell dysfunction after infusion. In the context of immunosenescence, a key consideration is that the quality of the T cells used for CAR transduction is influenced by donor age: T cells from older individuals exhibit shorter telomeres, reduced proliferative capacity, and a higher frequency of senescent cells. Selecting less differentiated T cell subsets or using gene-editing strategies to knock out senescence-associated genes may improve CAR T cell persistence and efficacy in aged hosts, but these approaches are at the preclinical stage.

The quality of the T cells used for CAR transduction is influenced by donor age: T cells from older individuals exhibit shorter telomeres, reduced proliferative capacity, and a higher frequency of senescent cells. Selecting less differentiated T cell subsets (e.g., naive or central memory T cells) or using gene-editing strategies to knock out senescence-associated genes (e.g., p16^INK4a, PD-1) may improve CAR T cell persistence and efficacy in aged hosts (53). Additionally, CAR T cells engineered to target senescent cell surface markers expressed on both tumor cells and senescent stromal cells could simultaneously achieve anti-tumor and senolytic effects (**Table 3**).

### 5.7 Combination strategies: integrating senotherapy with immunotherapy

The convergence of senotherapy and immunotherapy represents one of the most promising translational frontiers. Conceptually, the integration of these approaches addresses two complementary aspects of tumor immunosenescence: senolytics and senomorphics target the cellular source of SASP-mediated immune suppression, while immunotherapies activate or restore the effector arm of anti-tumor immunity. but remains largely untested in patients. The complexity of multi-agent regimens—involving choice of senescence inducer, senolytic agent, and immunotherapeutic backbone; dosing schedule; treatment sequence; and management of additive toxicities—poses substantial challenges for clinical development. The field currently lacks pharmacodynamic biomarkers that can measure on-target senolytic activity and immune restoration in real time. Moreover, whether the benefits of such complex combinations justify their toxicity and cost in older patients with pre-existing immunosenescence and limited physiological reserve is an open question.

Preclinical evidence supports the potential of such combinations. In doxorubicin-treated breast cancer models, the senolytic elimination of tumor cells combined with dual immune checkpoint blockade (anti-PD-1 plus anti-CTLA-4) produced superior tumor control compared with either approach alone (34). The combination of radiation, PARP inhibition (to induce senescence), navitoclax (to eliminate senescent cells), and ICI constitutes a multi-step regimen that addresses senescence induction, senescent cell clearance, and immune activation concurrently (38, 39).

For older patients with pre-existing immunosenescence, a rational treatment sequence may involve: (i) initial immune priming (e.g., STING agonist or low-dose cyclophosphamide) to enhance antigen presentation and T cell activation; (ii) concomitant or sequential ICI to amplify the effector T cell response; and (iii) intermittent senolytic dosing (“hit-and-run”) to eliminate therapy-induced senescent cells and prevent SASP-driven immune suppression and tumor relapse. Such protocols will require careful optimization of agent choice, dose, schedule, and sequence, guided by biomarkers that capture the dynamic state of the immune system in individual patients.

### 5.8 The breast microbiota, aging, and immune senescence

The microbiota—the community of microorganisms residing in breast tissue, the gut, and other body sites—plays a crucial role in modulating immune function and has emerged as a determinant of cancer development and therapeutic response. The breast microbiota differs from the gut microbiota in composition and diversity, and both undergo significant changes with age. Whether age-related shifts in the microbiota contribute to immunosenescence of the breast TME is a nascent but potentially important area of inquiry.

#### 5.8.1 The breast tissue microbiota

Contrary to the long-held belief that breast tissue is sterile, culture-independent sequencing has identified a distinct breast-resident microbiota dominated by Proteobacteria, Firmicutes, Actinobacteria, and Bacteroidetes. Breast cancer tissue harbors a microbiota compositionally distinct from adjacent normal tissue, with enrichment of specific taxa (e.g., Fusobacterium, Atopobium, Bacillus, Enterobacteriaceae). This tumor-associated microbiota may influence cancer biology through microbial metabolites that modulate estrogen metabolism, genotoxins that induce DNA damage, activation of pattern recognition receptors (TLRs, NLRs), and modulation of local cytokine and chemokine milieus.

#### 5.8.2 The gut-breast microbiota axis

The gut microbiota exerts systemic immunomodulatory effects through microbial metabolites (short-chain fatty acids, secondary bile acids) that influence immune cell function at distant sites, shaping of systemic innate immune tone through tonic TLR and NLR stimulation, promotion of DC maturation and T cell priming by specific bacterial species (Bifidobacterium, Akkermansia muciniphila), and modulation of systemic estrogen levels through the ‘estrobolome’. Age-related changes in gut microbiota composition (reduced diversity, expansion of pathobionts, loss of beneficial commensals) may contribute to both systemic immunosenescence and altered hormonal signaling in post-menopausal women.

#### 5.8.3 Microbiota and immune senescence: potential links

Although direct evidence linking the microbiota to immunosenescence in breast cancer is currently lacking, several plausible mechanistic connections exist: (i) age-related dysbiosis may reduce short-chain fatty acid production, which normally supports Treg homeostasis and systemic anti-inflammatory tone; (ii) chronic, low-grade microbial translocation across an aging gut epithelium may contribute to systemic inflammaging, which in turn drives immune cell senescence; (iii) the loss of beneficial immunomodulatory commensals with age may reduce tonic innate immune stimulation; (iv) microbial metabolites that modulate estrogen metabolism may influence hormonal effects on immune function; and (v) the breast-localized microbiota may directly modulate immune cell function within the TME.

#### 5.8.4 Therapeutic implications and knowledge gaps

Fecal microbiota transplantation (FMT) from young to aged mice has been shown to reverse some age-related immune phenotypes in preclinical models. Probiotic supplementation with specific immunomodulatory strains and dietary interventions are being explored as adjuncts to cancer immunotherapy. However, the field is characterized by several limitations: the low microbial biomass of breast tissue makes contamination a significant concern; many studies are correlative without establishing causality; the functional consequences of specific microbiota compositions are context-dependent; and commercial probiotic products are not regulated as drugs. Whether microbiota modulation can reverse or mitigate tumor immunosenescence in breast cancer patients is entirely unknown and represents a high-risk, high-reward research direction.

## 6 Conclusions and future directions

Tumor immunosenescence has emerged as a potentially important determinant of breast cancer progression and therapeutic resistance. The evidence reviewed here suggests that age-associated immune dysfunction may be an active participant in disease biology, operating through multiple interconnected mechanisms: (i) the accumulation of senescent and exhausted T cells—two mechanistically distinct states requiring different therapeutic approaches with impaired effector function and SASP-mediated bystander suppression; (ii) NK cell functional impairment driven by tumor-derived TGF-β and characterized by NKG2D downregulation; (iii) myeloid skewing toward immunosuppressive M2 macrophages and MDSCs;, amplified by bidirectional crosstalk with senescent CAFs(iv) diminished IFN signaling and antigen presentation with advancing age in a subset of patients(v) therapy-induced senescence as a paradoxical driver of immune evasion and disease relapse. Importantly, the mechanistic understanding of these processes derives predominantly from murine models and *in vitro* systems; the extent to which each mechanism operates in human breast cancer, and its quantitative contribution to patient outcomes, remains incompletely defined. We have further highlighted several breast cancer-specific dimensions of immunosenescence that warrant greater attention, including subtype-specific manifestations, hormonal and menopausal influences, stage-dependent immune alterations, BRCA-associated susceptibility, and the potential role of the breast and gut microbiota in modulating age-related immune dysfunction.

The clinical implications of these findings are substantial. but require validation in prospectively designed studies. Immunosenescence biomarkers—including TIL density (adjusted for age), IFN signaling signatures, p16^INK4a expression, and ISG^+^CD8^+^ T cell frequency—have the potential to refine prognostic stratification and guide the selection of immunotherapeutic regimens. However, all of these biomarkers face significant barriers to clinical implementation, including lack of consensus definitions, inter-laboratory variability, absence of multi-institutional validation, and the practical challenges of translating research-grade assays into clinical laboratory workflows. The demonstration that STING agonists can restore IFN signaling and ICI responsiveness in aged mice, and that NAD+ repletion can reverse metabolic senescence in CD8+ T cells in preclinical models, provides actionable therapeutic hypotheses that merit clinical evaluation. However, these hypotheses must be tested in rigorously designed trials before they can inform clinical practice.

Several critical knowledge gaps and research priorities emerge from this synthesis: First, the functional consequences of B cell and TLS immunosenescence in breast cancer are largely unexplored. Given the established role of TLS in promoting ICI responses, understanding whether TLS formation, maintenance, and function decline with age—and whether this contributes to reduced ICI efficacy in older patients—is a high-priority question. Second, the heterogeneity of senescent cell populations in the breast cancer microenvironment demands systematic characterization. Single-cell multi-omics approaches that simultaneously profile the transcriptome, epigenome, and proteome of individual senescent cells will be essential to delineate senescent subtypes, their SASP profiles, their immune evasion strategies, and their specific therapeutic vulnerabilities. Third, the clinical development of senotherapies in breast cancer will require biomarkers that identify patients most likely to benefit, measure on-target senolytic or senomorphic activity, and define optimal treatment schedules. The “hit-and-run” dosing paradigm—intermittent senolytic administration to eliminate senescent cells without continuous drug exposure—is clinically attractive but requires validation in adequately powered randomized trials. Fourth, the interplay between systemic immunosenescence and local tumor immune suppression is bidirectional and dynamic. Systemic interventions (e.g., exercise, caloric restriction mimetics, senolytics) that alleviate age-related immune dysfunction may have unexpected effects on the tumor microenvironment and vice versa. Studies that integrate systemic and tumor-intrinsic immune parameters across the treatment trajectory are needed. Fifth, clinical trials that specifically enroll older breast cancer patients and incorporate comprehensive immune monitoring are urgently needed. Most ICI trials have under-enrolled patients over 70 years of age, and the applicability of trial results to the typical breast cancer patient—who is diagnosed at a median age of 62—remains uncertain. Age-stratified trial designs and dedicated geriatric oncology trials with immune endpoints are essential to advance evidence-based care for older patients.

In conclusion, targeting tumor immunosenescence represents a conceptually compelling but still incompletely validated therapeutic axis in breast cancer. The convergence of fundamental insights into the biology of immune aging with clinical advances in senotherapy and immunotherapy creates an opportunity to develop age-adapted treatment strategies. However, realizing this opportunity will require a transition from descriptive, correlative studies toward mechanistically rigorous, prospectively designed investigations; the development and validation of consensus biomarkers for immunosenescence; the systematic incorporation of breast cancer-specific variables (subtype, menopausal status, disease stage, germline genetics) into study designs; and, most critically, randomized clinical trials that test whether targeting immunosenescence improves outcomes for the breast cancer patients whose disease is shaped by the biology of aging.

## Glossary

ABC: age-associated B cell
ACT: adoptive cell therapy
AMPK: AMP-activated protein kinase
CAF: cancer-associated fibroblast
CAR: chimeric antigen receptor
CCL: C-C motif chemokine ligand
CDK: cyclin-dependent kinase
CTLA-4: cytotoxic T-lymphocyte-associated protein 4
CXCL: C-X-C motif chemokine ligand
DC: dendritic cell
DAMP: damage-associated molecular pattern
DNAM-1: DNAX accessory molecule 1
EMT: epithelial-mesenchymal transition
FOXP3: forkhead box P3
HER2: human epidermal growth factor receptor 2
HGF: hepatocyte growth factor
HR: hormone receptor
ICI: immune checkpoint inhibitor
IFN: interferon
IL: interleukin
iNOS: inducible nitric oxide synthase
IRF9: interferon regulatory factor 9
JAK: Janus kinase
KIR: killer immunoglobulin-like receptor
LAG-3: lymphocyte activation gene 3
M-MDSC: monocytic myeloid-derived suppressor cell
MHC: major histocompatibility complex
MICA/B: MHC class I chain-related protein A/B
MMP: matrix metalloproteinase
MDSC: myeloid-derived suppressor cell
NAD: nicotinamide adenine dinucleotide
NF-κB: nuclear factor kappa-light-chain-enhancer of activated B cells
NK: natural killer
NKG2A: natural killer group 2 member A
NKG2D: natural killer group 2 member D
NLRP3: NOD-, LRR- and pyrin domain-containing protein 3
NMN: nicotinamide mononucleotide
PARP: poly (ADP-ribose) polymerase
PD-1: programmed cell death protein 1
PD-L1: programmed death-ligand 1
PMN-MDSC: polymorphonuclear myeloid-derived suppressor cell
PPAR: peroxisome proliferator-activated receptor
ROS: reactive oxygen species
SA-β-gal: senescence-associated beta-galactosidase
SASP: senescence-associated secretory phenotype
STAT: signal transducer and activator of transcription
STING: stimulator of interferon genes
TAM: tumor-associated macrophage
TCR: T cell receptor
TGF-β: transforming growth factor beta
Th: T helper
TIGIT: T cell immunoreceptor with Ig and
TIL: tumor-infiltrating lymphocyte
TIM-3: T cell immunoglobulin and mucin
TIS: therapy-induced senescence
TLS: tertiary lymphoid structure
TME: tumor microenvironment
TNF-α: tumor necrosis factor alpha
Treg: regulatory T cell
VEGF: vascular endothelial growth factor
